# Multi-Omics Association Analysis of DOF Transcription Factors Involved in the Drought Resistance of Wheat Induced by *Strigolactone*

**DOI:** 10.3390/ijms26062396

**Published:** 2025-03-07

**Authors:** Yanjing Wang, Haiyang Jin, Simeng Du, Baoting Fang, Junqin Yue, Cheng Yang, Hanfang Wang, Deqi Zhang, Jiarui Wang, Hang Song, Yunhui Shao, Xiangdong Li

**Affiliations:** 1 Wheat Research Institute, Henan Academy of Agricultural Sciences, Zhengzhou 450002, China; 2 College of Life Sciences, Zhengzhou Normal University, Zhengzhou 450044, China

**Keywords:** drought, wheat, *Strigolactone*, transcription factors, multi-omics analysis

## Abstract

Drought is one of the main adverse factors affecting the growth and development of wheat. The molecular regulation pathway of *Strigolactone* (SLs or SL),which induces drought resistance in wheat, needs to be further clarified. In this study, SL and Tis (*Strigolactone* inhibitor) were sprayed on leaves to clarify the changes in wheat drought resistance and their effect on antioxidant enzyme activity, photosynthesis and other metabolic processes. However, 20 kinds of DOF transcription factors were identified by transcriptome metabolome association analysis, and they were highly enriched on chromosome 2. Moreover, the proline, glycosides, indoleacetic acid, betaine, etc., in wheat are the key factors affecting the change in the drought resistance of wheat. The study initially revealed the mechanism of the involvement of DOF in the SL regulation pathway and revealed its impact on different metabolites of wheat, thus providing a theoretical reference for the subsequent molecular verification and breeding of excellent drought-resistant varieties.

## 1. Introduction

Precipitation plays a pivotal role in influencing global agricultural production, particularly grain cultivation in underdeveloped nations. As global temperatures climb, extreme weather events have become more prevalent, with droughts posing a severe threat to grain production and supply in these countries. Wheat stands out not only as one of the world’s three primary grain crops but also as a staple cultivated in arid regions. According to statistics from the United Nations Food and Agriculture Organization, wheat cultivation spans 216 million hectares worldwide, yielding an average of 3.5 tons per hectare for a total output of 765 million tons [1]. This crucial crop is a major food import for underdeveloped regions like Africa.

Considering the current state of global socio-technological development, rising temperatures are projected to decrease global wheat production by 1.9% by mid-century due to extreme weather. Notably, tropical African and South Asian underdeveloped countries will experience declines of approximately 15% and 16%, respectively [2]. Furthermore, studies indicate that in key wheat-producing areas, high temperatures and rainfall patterns impact wheat yield formation by over 40% [3]. Wheat cultivation predominantly occurs in temperate regions, which unfortunately coincide with areas prone to droughts over the past decade [1]. Therefore, enhancing wheat’s drought resistance and fortifying its supply are of utmost importance to safeguard global food security and socio-economic stability.

Plants effectively address water scarcity by regulating their hormonal metabolism, which in turn influences phenotypic and physiological metabolic changes. Research has demonstrated that plants can boost their water use efficiency through various mechanisms such as increasing root hair density [4], closing stomata [5], elevating carbohydrate and other compound levels [6,7], enhancing antioxidant enzyme activity, and other strategies [8]. These adaptations ensure the smooth progression of vital growth and developmental processes like photosynthesis [9]. However, these activities are influenced by alterations in plant hormone metabolism [10].

Drought conditions diminish water use efficiency and stimulate the production of abscisic acid (ABA), which controls stomatal closure, enhances photosynthetic capacity, improves osmotic adjustment, and bolsters plant drought tolerance [11]. Upon receiving stress signals, the cell plasma membrane converts lutein into ABA and releases it. Most ABA is synthesized in the root system and transported upward through vascular tissue [12,13]. ABA receptors, widely present in organelles like the nucleus, cytoplasm, and chloroplast, inhibit the action of sucrose non-fermenting 1-related protein kinase 2 (SnRK2) proteins via protein phosphatase 2C (PP2C) under low ABA concentrations, leading to dephosphorylation and regulating cellular drought stress metabolism [14].

Cytokinin and auxin are crucial hormones for plant growth, cell division and proliferation, organ formation, and material and energy metabolism, playing significant roles throughout the plant life cycle [15,16,17]. RIE Nishiyama et al. [18] directly showed that cytokinin (CK) negatively regulates salt and drought stress signals in Arabidopsis mutants. CK-deficient Arabidopsis exhibits strong stress tolerance due to increased cell membrane integrity and ABA hypersensitivity. Under normal conditions, while CK deficiency heightens plant sensitivity to exogenous ABA, it downregulates key ABA biosynthetic genes, resulting in significantly lower endogenous ABA levels in CK-deficient plants compared to wild-type plants. Research has indicated that auxin homeostasis modulates ABA production and the drought stress response [19]. Drought significantly reduces the transcript abundance of indole-3-acetic acid (IAA) synthesis genes in rice (*Oryza sativa* L.) and elevates the transcript abundance of IAA-conjugate genes [20].

Gibberellin (GA) is involved in regulating rice seed germination, stem elongation, and reproductive development under drought conditions [21]. Changes in GA synthesis genes can interact with other hormones, influencing various plant growth and developmental processes [22]. As a secondary metabolic phenolic molecule, salicylic acid (SA) not only regulates plant carbon dioxide assimilation, antioxidants, stomatal closure, and photosynthesis [23] but also controls the stomatal aperture by modulating drought-related gene expression [24,25,26]. Plants overexpressing the SA synthesis transcription regulator CBP60g gene are more sensitive to ABA and exhibit enhanced drought tolerance [27].

In water-deficit conditions, although JAZ proteins are degraded, leading to the activation of transcription factors like MYC2 and the upregulation of related drought resistance genes [28], exogenous jasmonic acid (JA) application can regulate plant stomatal dynamics and improve plant growth [29]. Additionally, studies have shown that drought tolerance is reduced in brassinosteroid (BR)-deficient cotton mutants [30], while it is enhanced in etol1 rice mutants that accumulate more ethylene [31].

*Strigolactone*, a derivative of carotenoids, plays a pivotal role in plant development, influencing the formation of roots, leaves, and branches. Additionally, it promotes the germination of arbuscular mycorrhizal fungi in roots and has been extensively studied for its ability to regulate plant stress resistance [32,33,34]. However, natural Strigolactone (SL) levels are low in many plants, prompting the chemical synthesis of SL analogs such as GR5, GR7, and GR24, with GR24 exhibiting the highest activity [35].

Osmotic stresses, including salinity and drought, negatively impact the production of Tri-hydroxylactone in dicotyledonous plants like tomato, lettuce, and Lotus Japonicus [36,37]. Prior research has demonstrated that SL can mitigate drought stress in wheat by enhancing cell wall formation and optimizing root structure [38]. Specifically, during the mid to late growth stages of wheat under drought stress, SL reduces membrane lipid peroxidation in canopy leaves and boosts the plant’s osmotic adjustment capabilities by increasing antioxidant enzyme activity. This ensures smooth photosynthesis and stable yield formation [39,40].

Current studies, leveraging multi-omics technology, further clarify that exogenous GR24 can upregulate the expression or synthesis of drought-resistant molecules and metabolites in grapes [34], barley [41], and corn [42]. While we have gained considerable insights into SL’s role in regulating plant drought resistance, the exact regulatory pathway remains elusive, particularly the intricate cross-response mechanisms between SL and other hormones. These areas require further exploration and clarification.

Gene transcription regulation plays a crucial role in plant growth metabolism, hormone signal transduction, stress response, and various other biological processes. The DOF (DNA binding with one finger) family of transcription factors, unique to plants, is characterized by a distinctive single zinc finger structure. With recent advancements in species classification, numerous DOF members have been identified as key players in the plant life cycle.

These DOF proteins are involved in regulating a wide array of plant biological processes, including dormancy, tissue differentiation, carbon and nitrogen assimilation, and carbohydrate metabolism. Furthermore, they have been reported to play a role in modulating hormone signals and responding to both biotic and abiotic stresses.

CDF, a member of the DOF transcription factor family, is extensively involved in plant responses to various abiotic stresses. For instance, the mutant cdf3-1 gene enhances Arabidopsis sensitivity to drought and cold stress, while its overexpression unexpectedly boosts plant resistance to osmotic stress [43]. In wheat, the DOF protein taznf promotes downstream gene expression, leading to increased Na+ excretion and enhanced salt resistance [44]. Under long-term drought conditions, the overexpression of the woody apple DOF family gene mddof54 results in higher photosynthetic rates and branch water-carrying capacity compared to wild-type plants, while its survival rate under short-term drought conditions is significantly improved [45].

DOF proteins also participate widely in plant stress responses by responding to plant hormone signals. In castor, many rcdof proteins exhibited differential expression levels under ABA treatment [46]. Similarly, researchers have suggested that CmDOFs in chrysanthemum may be involved in the response to ABA and SA, leading to distinct expression patterns. Specifically, exogenous ABA significantly upregulated the expression levels of CmDOF12 and CmDOF20, while SA upregulated the expression levels of CmDOF2, CmDOF5, CmDOF6, CmDOF10, and CmDOF12 [47].

Although some studies have investigated the mechanism of DOF’s involvement in plant hormone response regulation, research on the mechanism of DOF’s involvement in Strigolactone (SL) response regulation remains to be further conducted.

## 2. Results

### 2.1. Morphological and Physiological Changes

**Figure 1:**
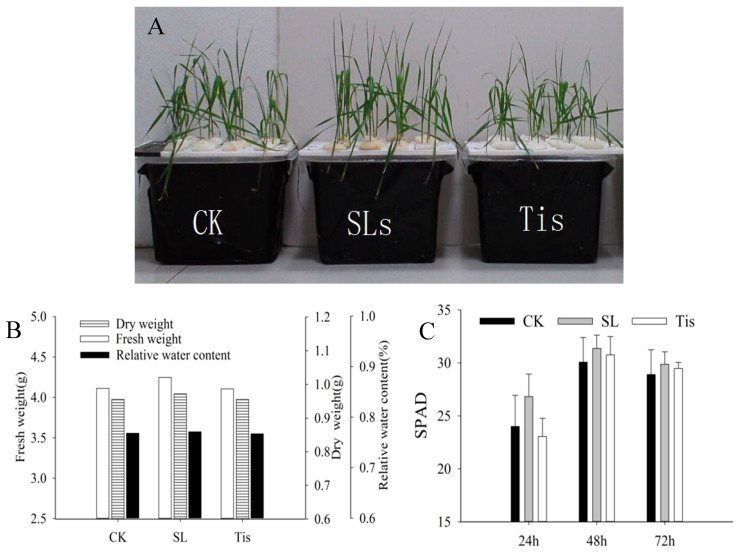
Morphological changes. Remarks: CK indicates control, SLs indicates unicorn lactone treatment, and Tis indicates inhibitor ((**A**) changes in wheat canopy morphology; (**B**) fresh weight, dry weight, and moisture content of plants; (**C**) leaf SPAD).

After 72 h of treatment, through visual observation, the results show that the growth state of wheat canopy leaves at the seedling stage after SL treatment was better than that of CK. On the other hand, the wheat canopy for the Tis treatment group was significantly inhibited, and the degree of leaf curling and wilting was the largest in drought conditions (Figure 1A). In contrast, the relative water content, dry matter accumulation and fresh weight of wheat in the different treatments were SLs > CK > Tis (Figure 1B). Moreover, the SPAD value for the different treatments was the highest in the SL treatment group, and it was higher in the different treatments at 48 h than at 24 and 72 h (Figure 1C).

**Figure 2:**
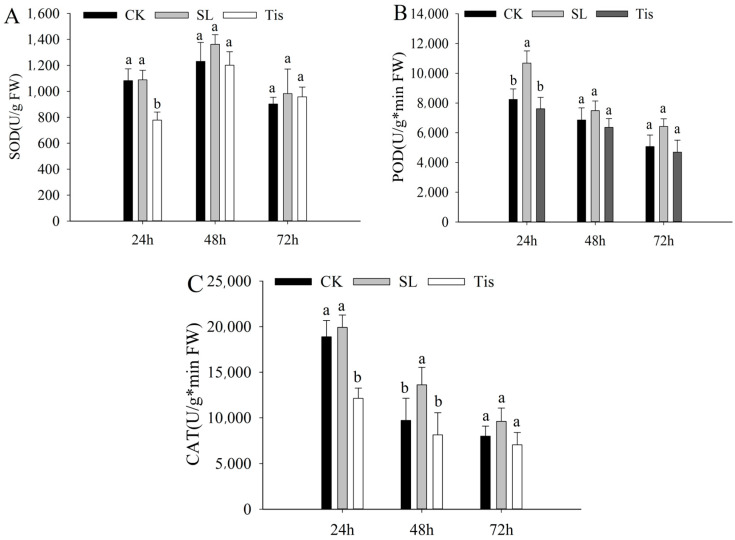
Physiological changes. Remarks: CK indicates control, SL indicates Strigolactone treatment, and Tis indicates inhibitor. The difference is significant (*p* < 0.05) and is indicated by the letters a and b ((**A**) superoxide dismutase activity; (**B**) peroxidase activity; (**C**) catalase activity).

Figure 2 shows the changes in three antioxidant enzyme activities in the canopy leaves. Among the three treatments, the SOD activity (Figure 2A) was higher at 48 h than that at 24 h and 72 h, but the POD and CAT activities (Figure 2B,C) decreased with time. As shown in the figures, there were significant differences in the changes in the SOD and POD activities at 24 h and CAT activities at 24 h and 48 h. The differences in other time periods were significant, and the enzyme activities of SL were the highest in the different treatments, which showed that compared with the control, the SL treatment could effectively improve the antioxidant enzyme activities of canopy leaves, while Tis could not, and the effects of the agents were mostly concentrated within 48 h.

### 2.2. Transcriptome Analysis and Transcription Factor Screening and Localization

**Figure 3:**
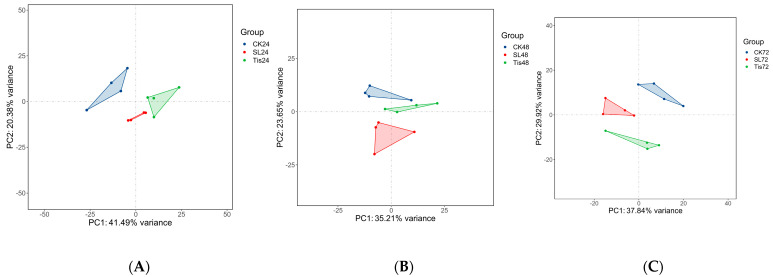
Transcriptome PCA. ((**A**) the 24 h treatment; (**B**) the 48 h treatment; (**C**) the 72 h treatment).

Figure 3 shows the correlation of different RNA-seq analyses among different treatments at 24 h, 48 h, and 72 h. RNA-seq was performed on leaves with four biological replicates of each treatment. A total of 36 samples obtained 2.821 Gb clean reads, with a Q30 base percentage of 91.94% and above and an average GC content of 53.40%, which met the sequencing requirements. By aligning reads to the reference genome, valid bases ranged from 92.95 to 97.38%. The data quality is reliable. The clustering of groups into different periods was clear, and the clustering within groups was close.

**Figure 4:**
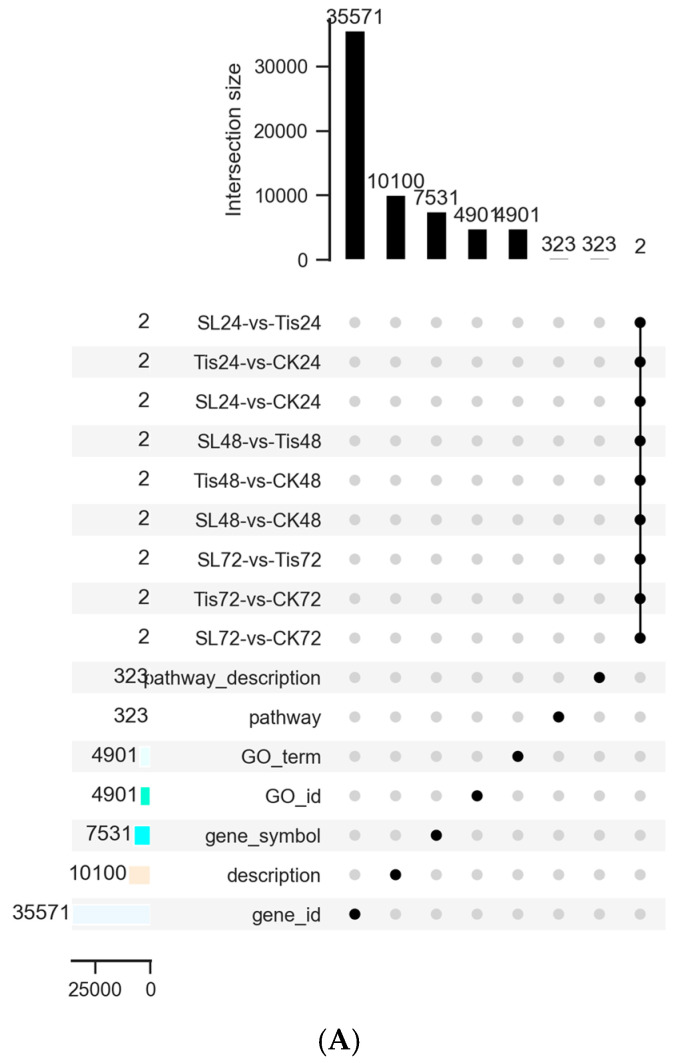

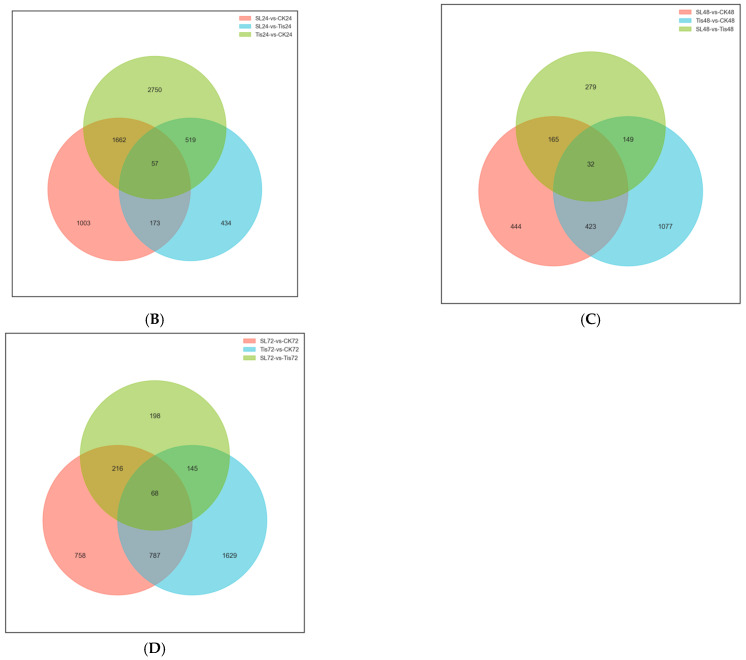
Transcriptome Venn diagram ((**A**) Venn diagram column chart; (**B**) the 24 h Venn diagram pie chart; (**C**) the 48 h Venn diagram pie chart; (**D**) the 72 h Venn diagram pie chart).

**Figure 5:**
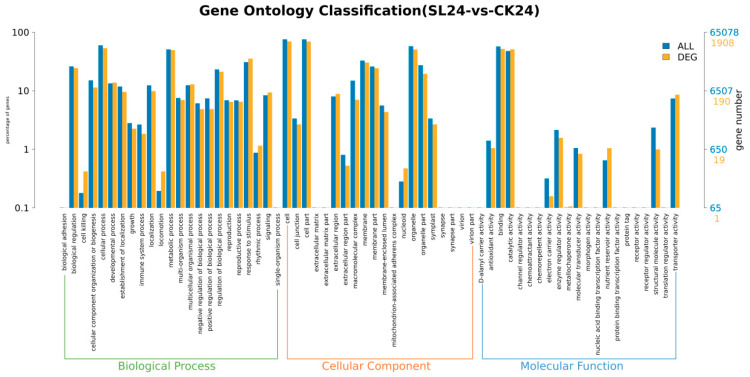

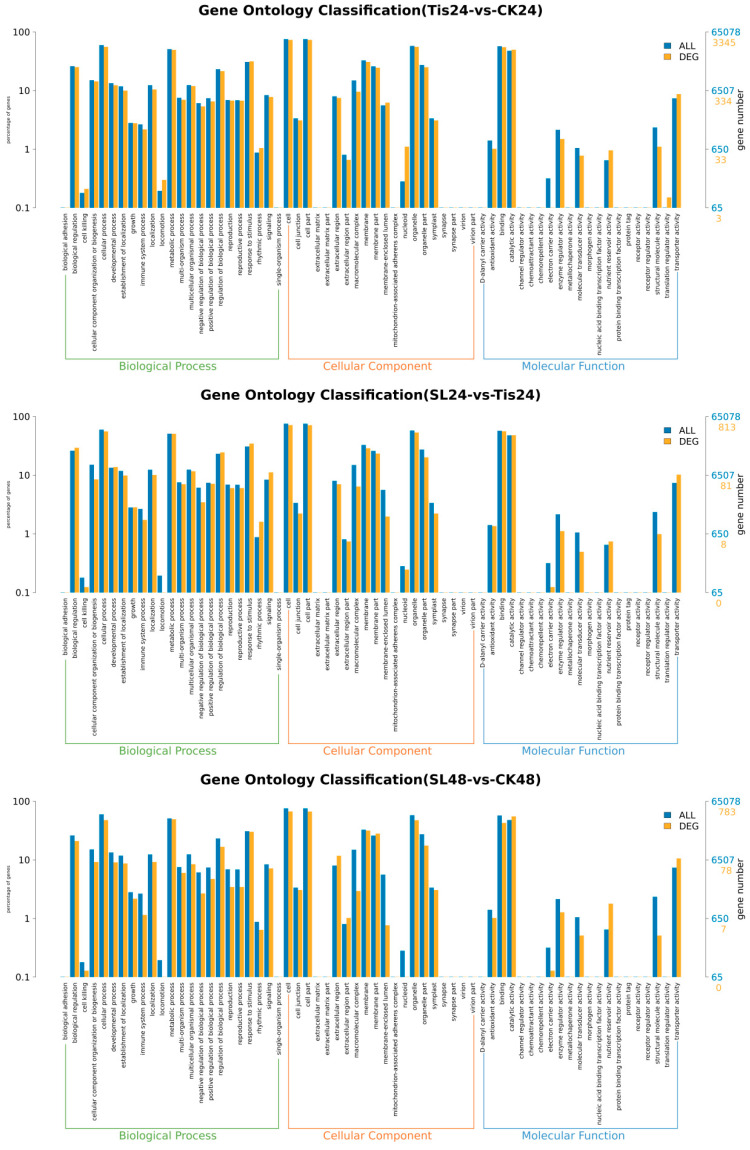

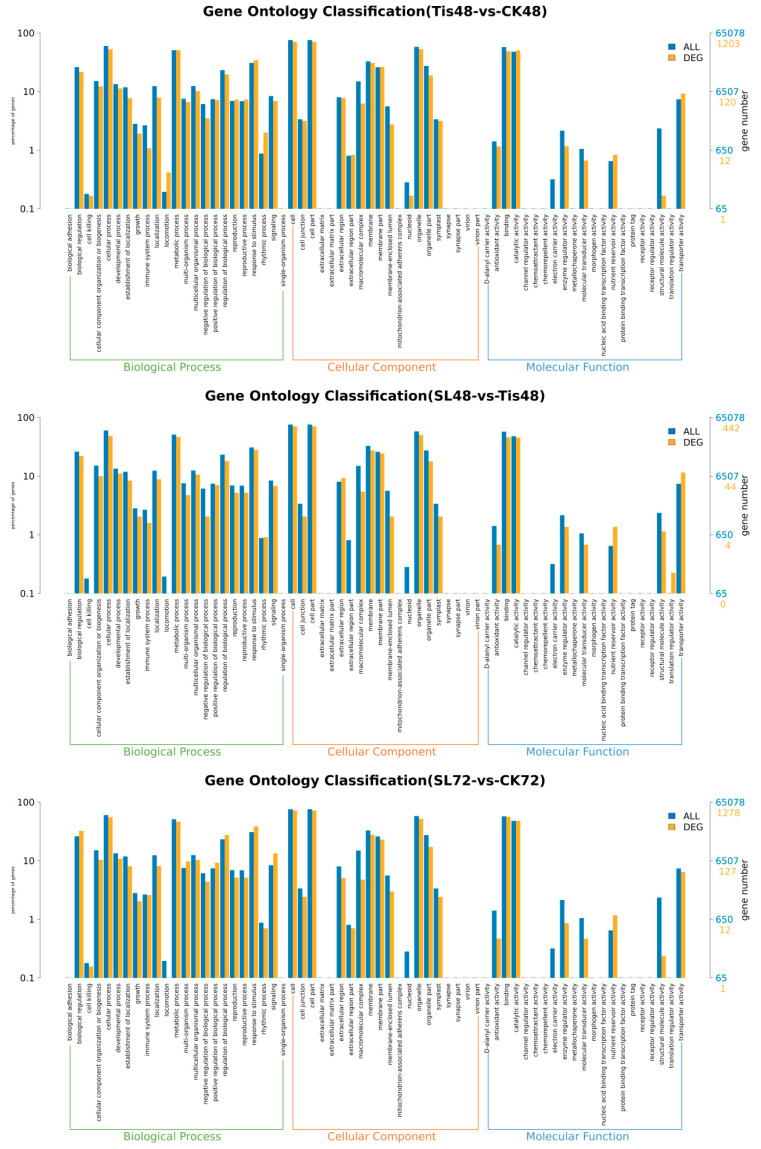

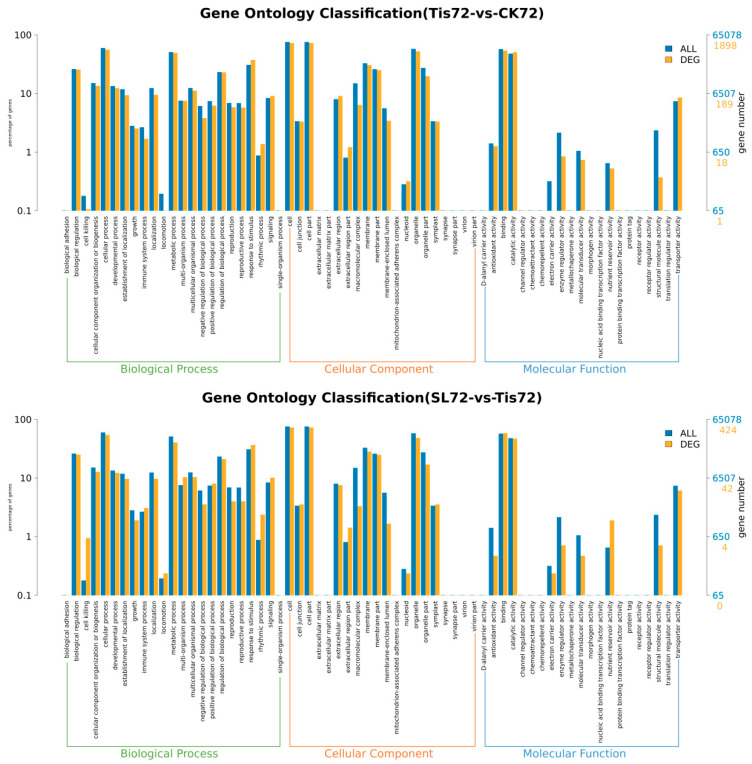
Comparison of transcriptome differentially expressed genes and distribution of all genes at GO Level 2. Remarks: 24, 48, and 72 represent the sampling time after treatment with different drugs, SL represents treatment with *Strigolactone*, Tis represents treatment with *Strigolactone* inhibitor, and CK represents control treatment.

As shown in Figure 4 and Figure 5, fold change (FC) ≥ 1 and FDR < 0.05 were used as the screening criteria for DEGs. A total of 65,078 genes were screened by comparing different treatments at different times: 2895 (SL24 vs. ck24), 1183 (SL24 vs. Tis24), 4988 (Tis24 vs. ck24), 1064 (SL48 vs. ck48), 625 (SLs48 vs. Tis48), 1681 (Tis48 vs. ck48), 1829 (SL72 vs. ck72), 627 (SL72 vs. Tis72), and 2629 (Tis72 vs. ck72).

**Figure 6:**
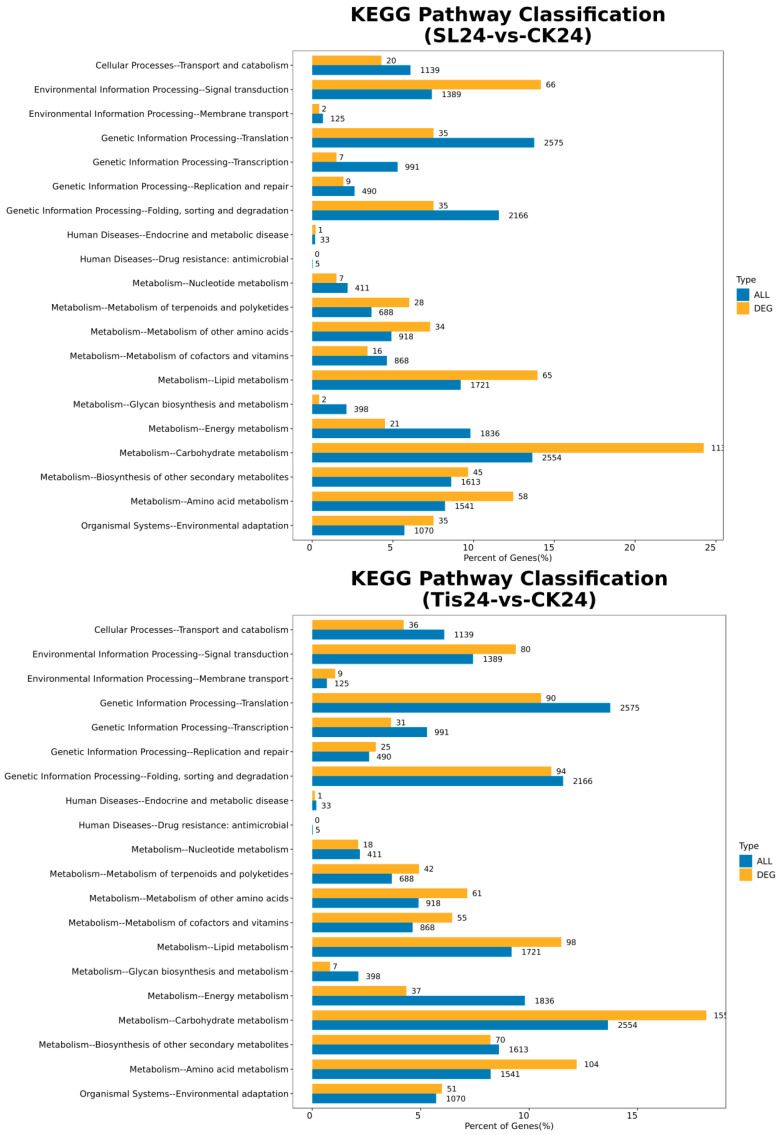

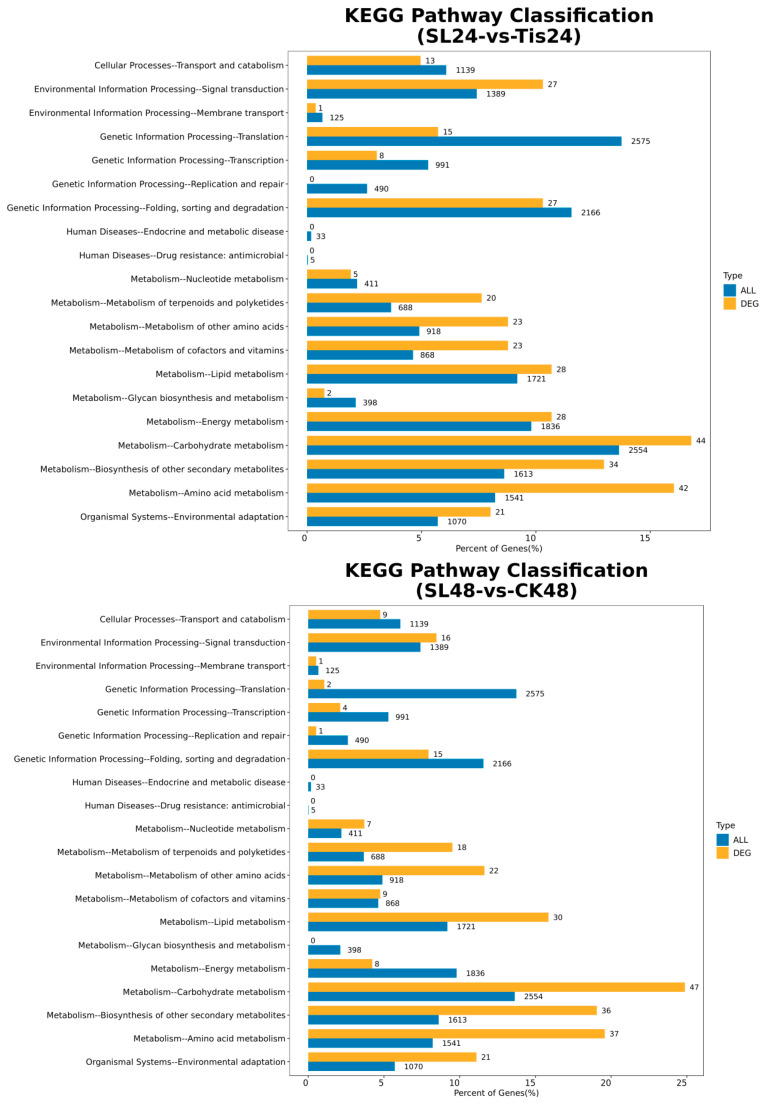

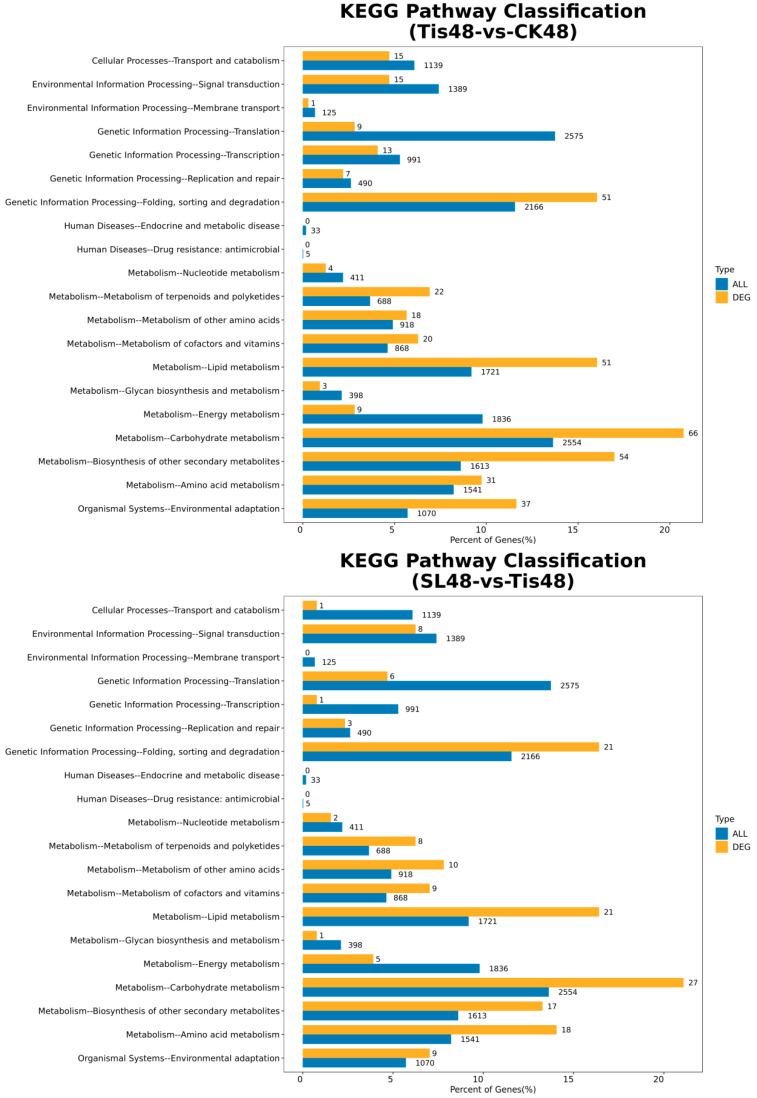

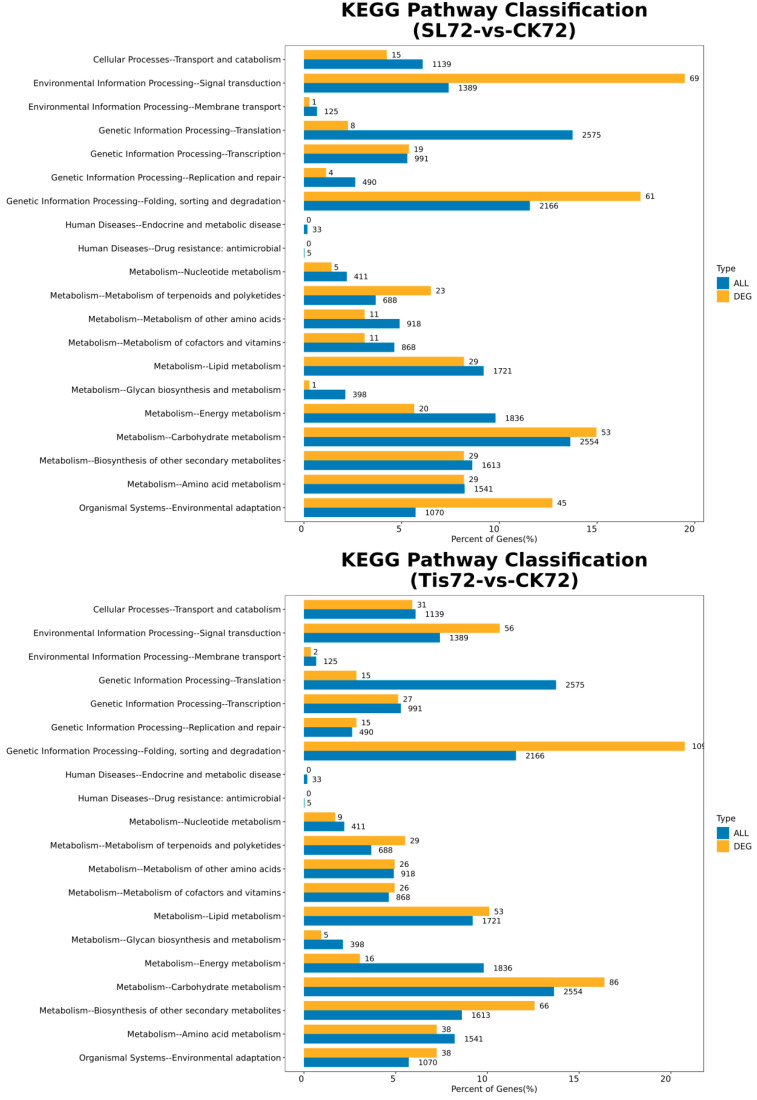

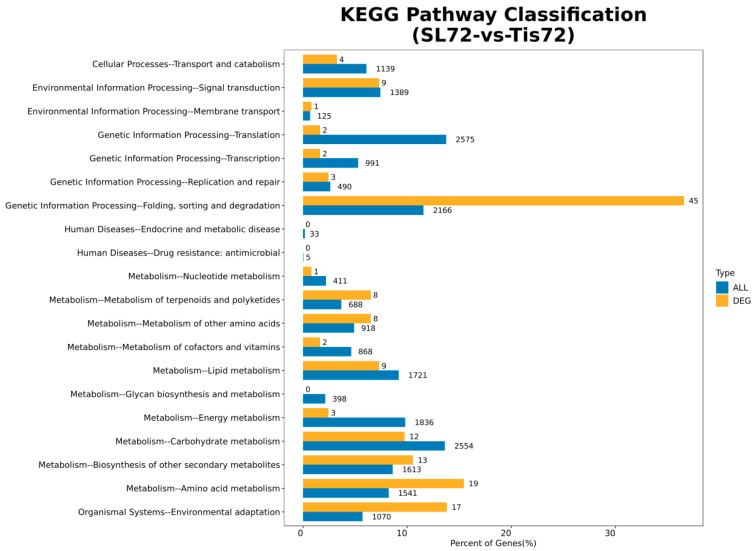
Comparison of transcriptome differentially expressed genes and distribution of all genes at KEGG Level 2. Remarks: 24, 48, and 72 represent the sampling time after treatment with different drugs, SL represents treatment with *Strigolactone*, Tis represents treatment with *Strigolactone* inhibitor, and CK represents control treatment.

As shown in Figure 5 and Figure 6, Go Functional Annotation was performed on all DEGs. The SL and Tis treatments had similar effects on wheat cell components, biological processes, and molecular functions. Among the different treatments, the impact on molecular function involves antioxidant metabolism, carbon metabolism, transcriptional regulation, and the activation of substance transport. In terms of cellular composition, different treatments mainly affect the stability of the membrane lipid structure and the regulation of the organelle structure and function in wheat leaf cells. It should be noted that more genes are expressed in response to external stimuli, ensuring cell stability and cellular function. In addition, according to the KEGG second-level distribution of differentially expressed genes, there are many differentially expressed genes in wheat carbon metabolism affected by exogenous SL and Tis, and there are also differences in genes involved in signal transduction.

**Figure 7:**
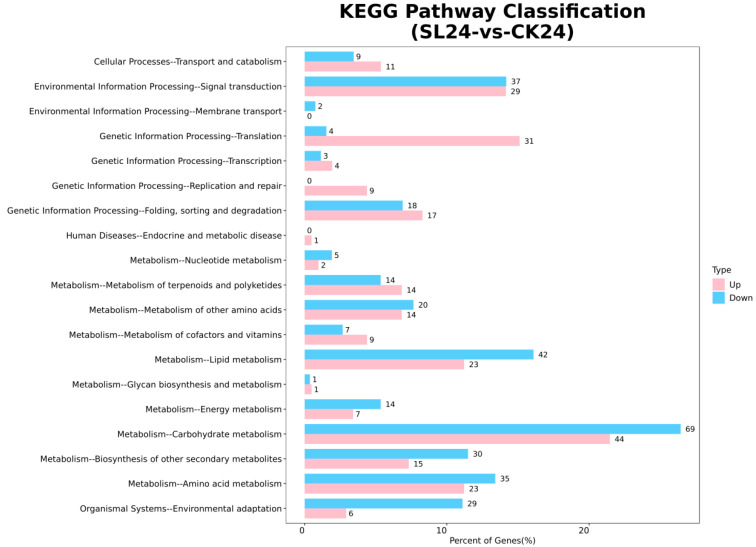

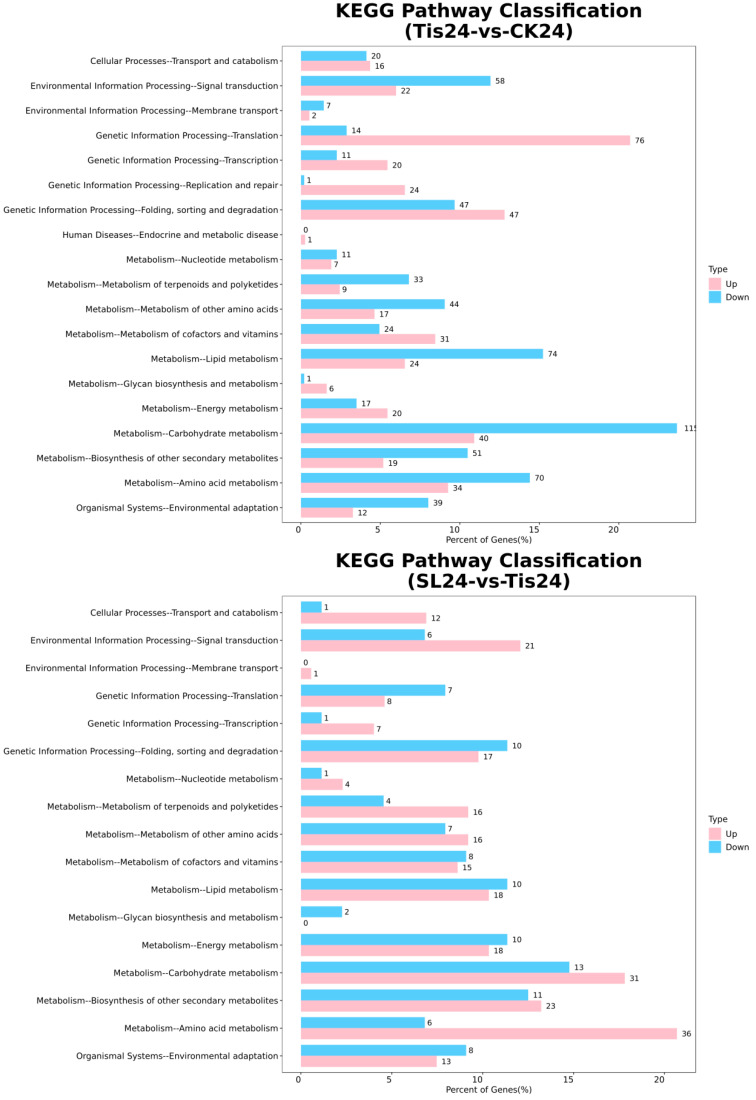

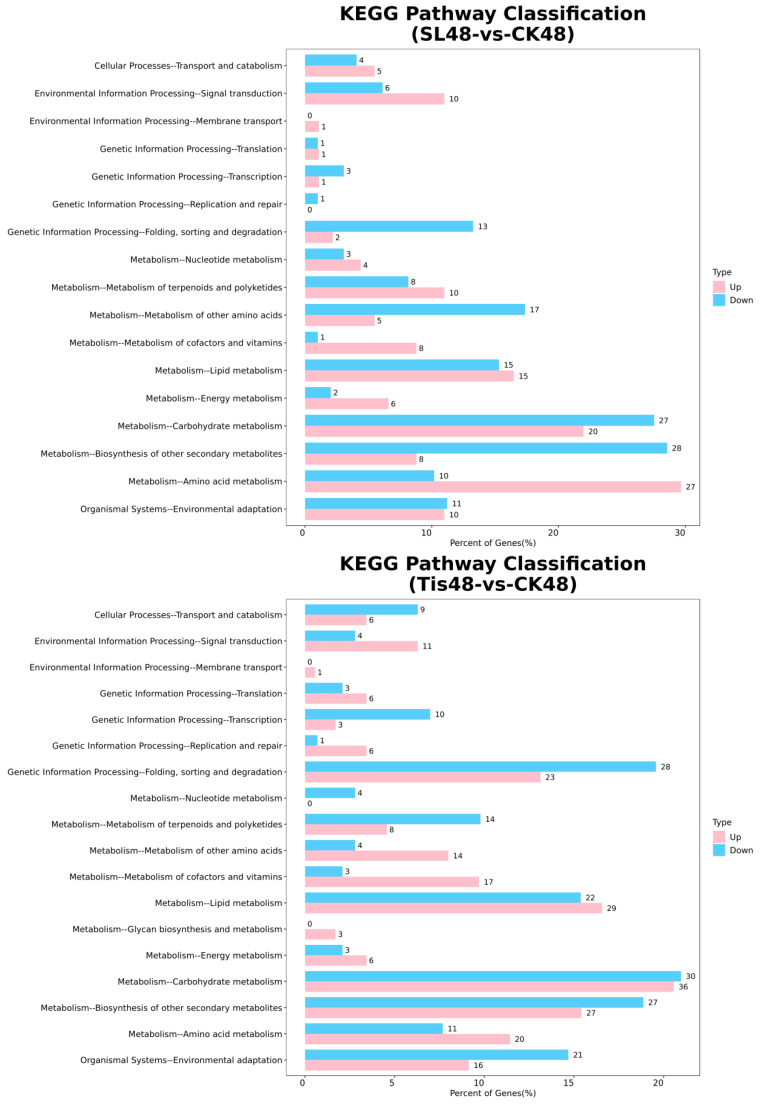

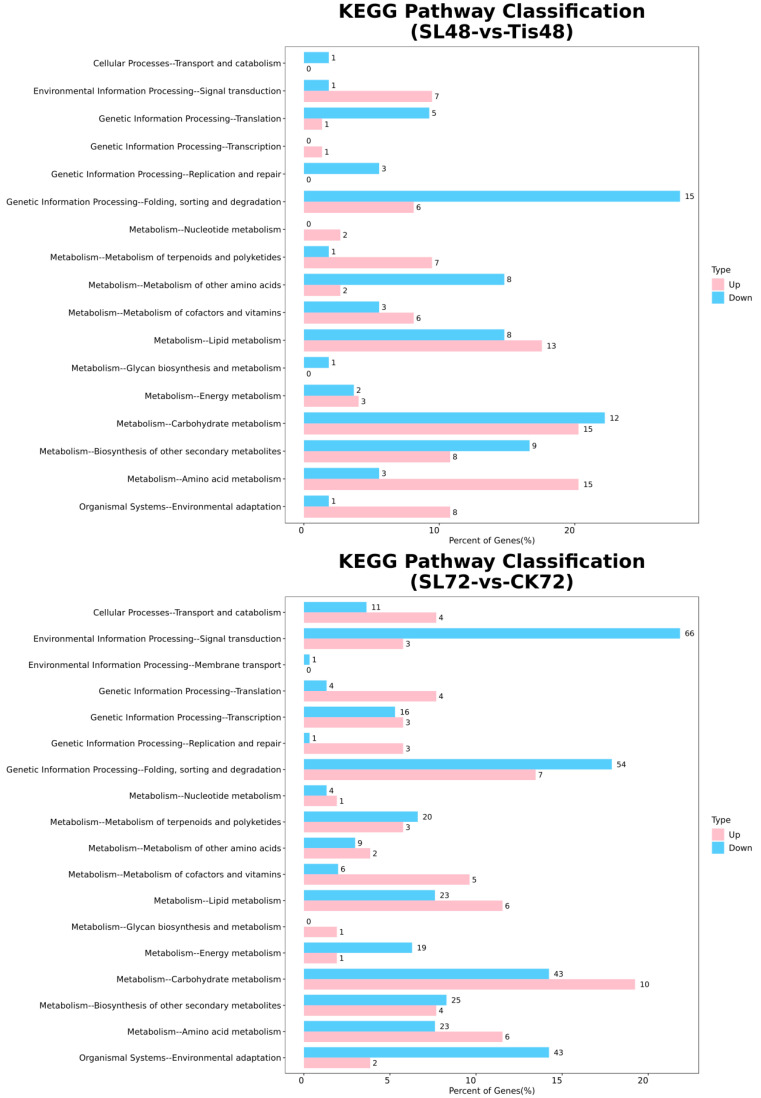

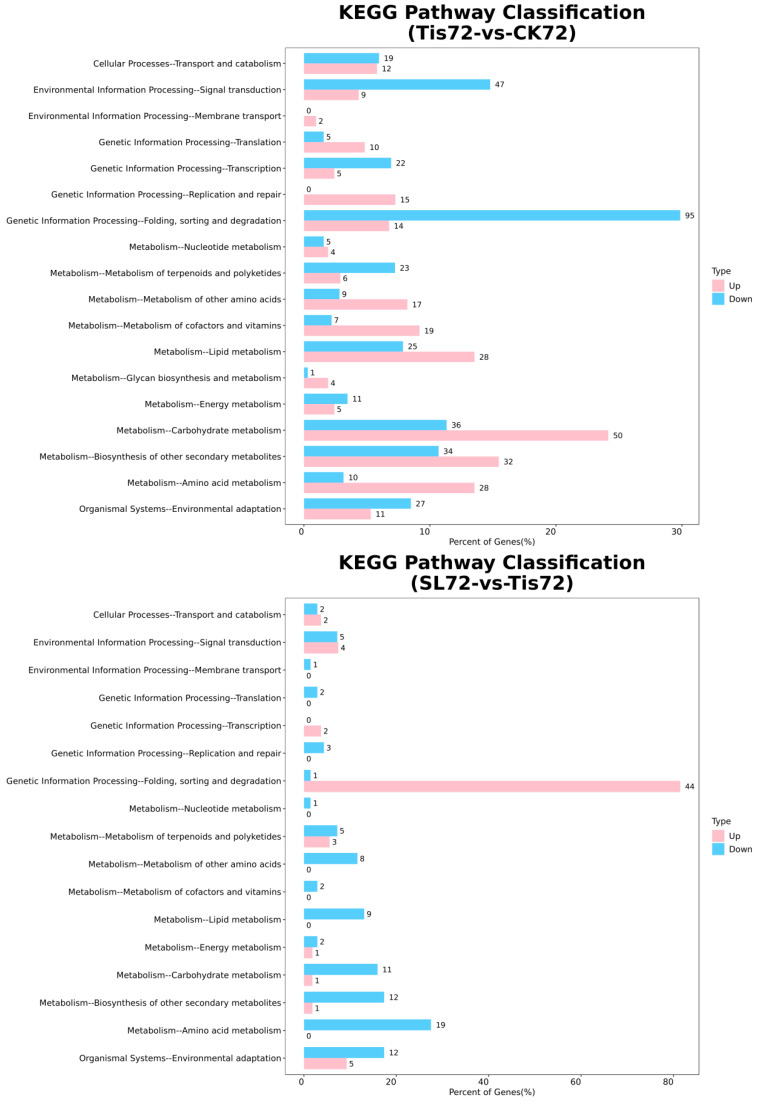
Distribution of upregulated and downregulated differentially expressed genes in transcriptome at KEGG Level 2. Remarks: 24, 48, and 72 represent the sampling time after treatment with different drugs, SL represents treatment with *Strigolactone*, Tis represents treatment with *Strigolactone* inhibitor, and CK represents control treatment.

As shown in Figure 7, the cluster analysis of all differential genes showed that at 24 and 48 h, there were more differential genes in signal transduction and carbon metabolism, while at 72 h, the number of differential genes in signal transduction decreased, while the number of synthetic genes involved in maintaining the stable metabolites of cells such as carbon metabolism and amino acid metabolism increased. This shows that the signal transduction effects of the two exogenous substances on drought resistance in wheat mainly appear within 24 and 48 h, after which the cells begin to enter a stable period due to the changes in the synthesis of their own substances.

As shown in Table 1, through the transcription factor analysis of sequencing data, a total of 20 kinds of DOF transcription factors (DOF TFs) were found, and their localization analysis was carried out. Most of the DOF TFs were concentrated on chromosomes 1, 2, 3, 5, and 6. Among them, a, B, and D of chromosome 2 were well distributed, and the number was large.

### 2.3. Metabolome Analysis

**Figure 8:**
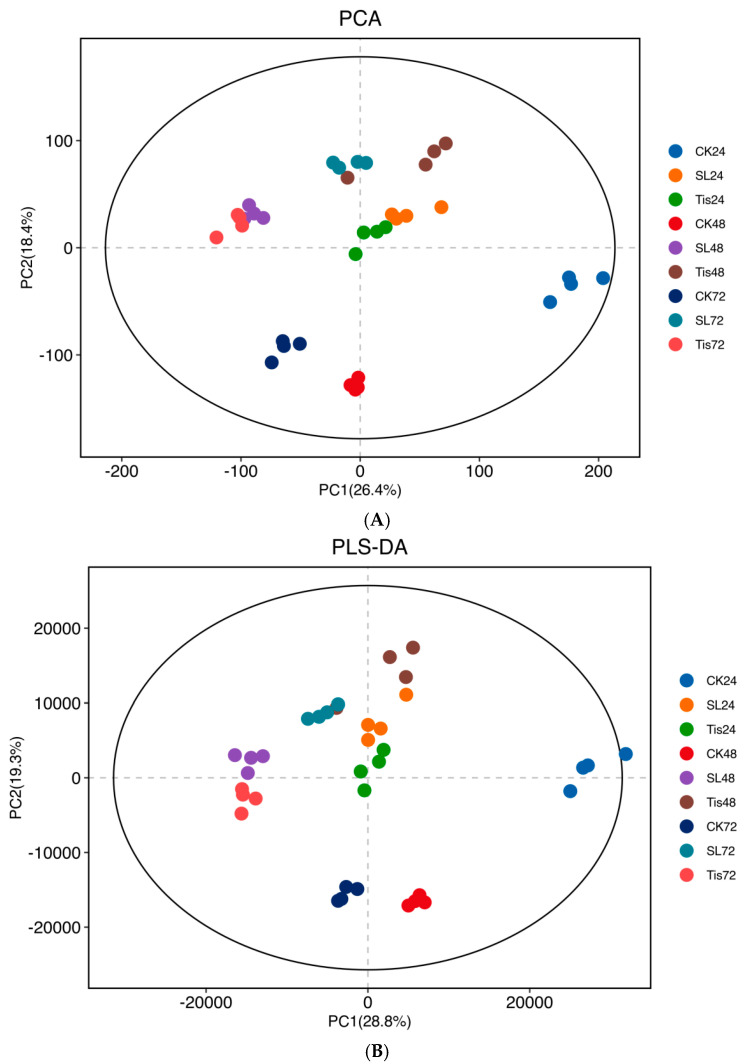
Metabolomics relevance. Remarks: (**A**) PCA; (**B**) PLS-DA. CK24/SL24/Tis24 were the 24 h treatments. CK48/SL48/Tis48 were the 48 h treatments. CK72/SL72/Tis72 were the 72 h treatments.

**Figure 9:**
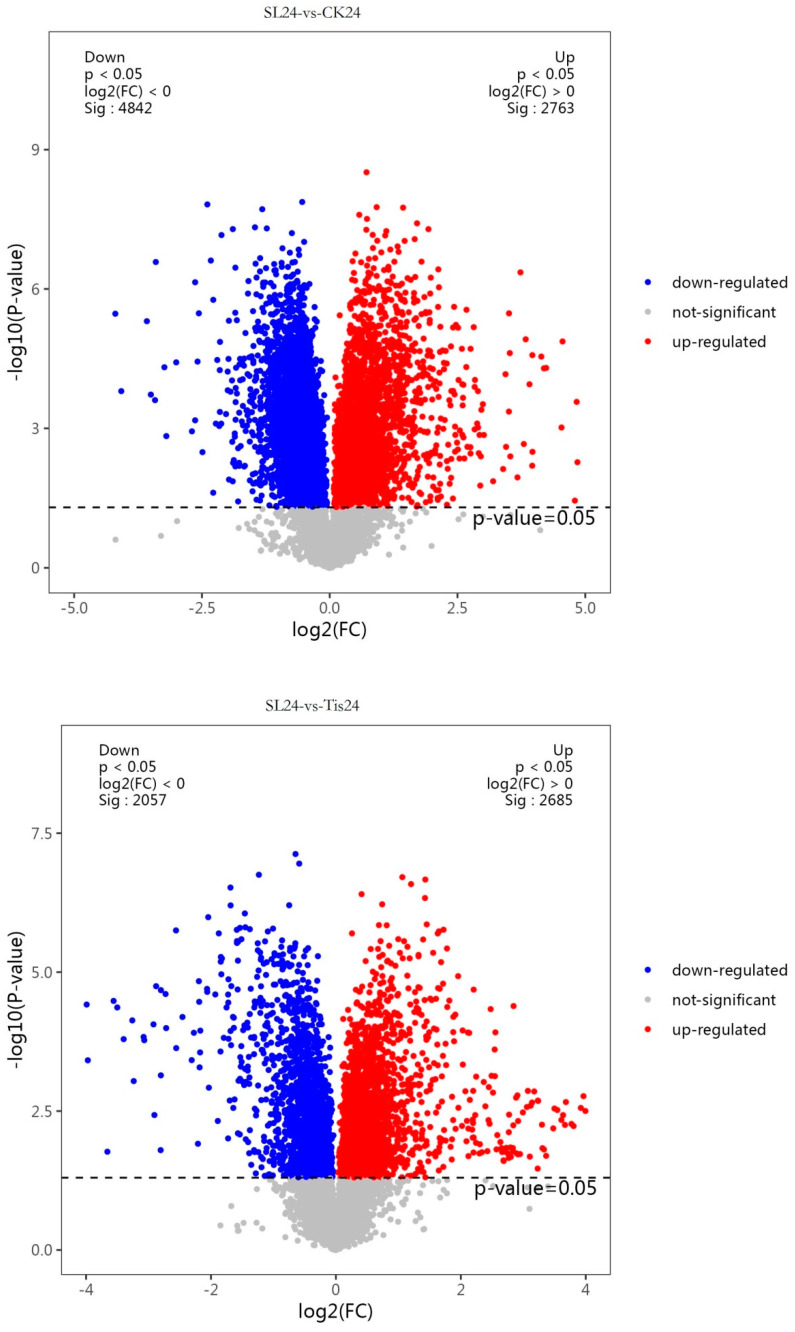

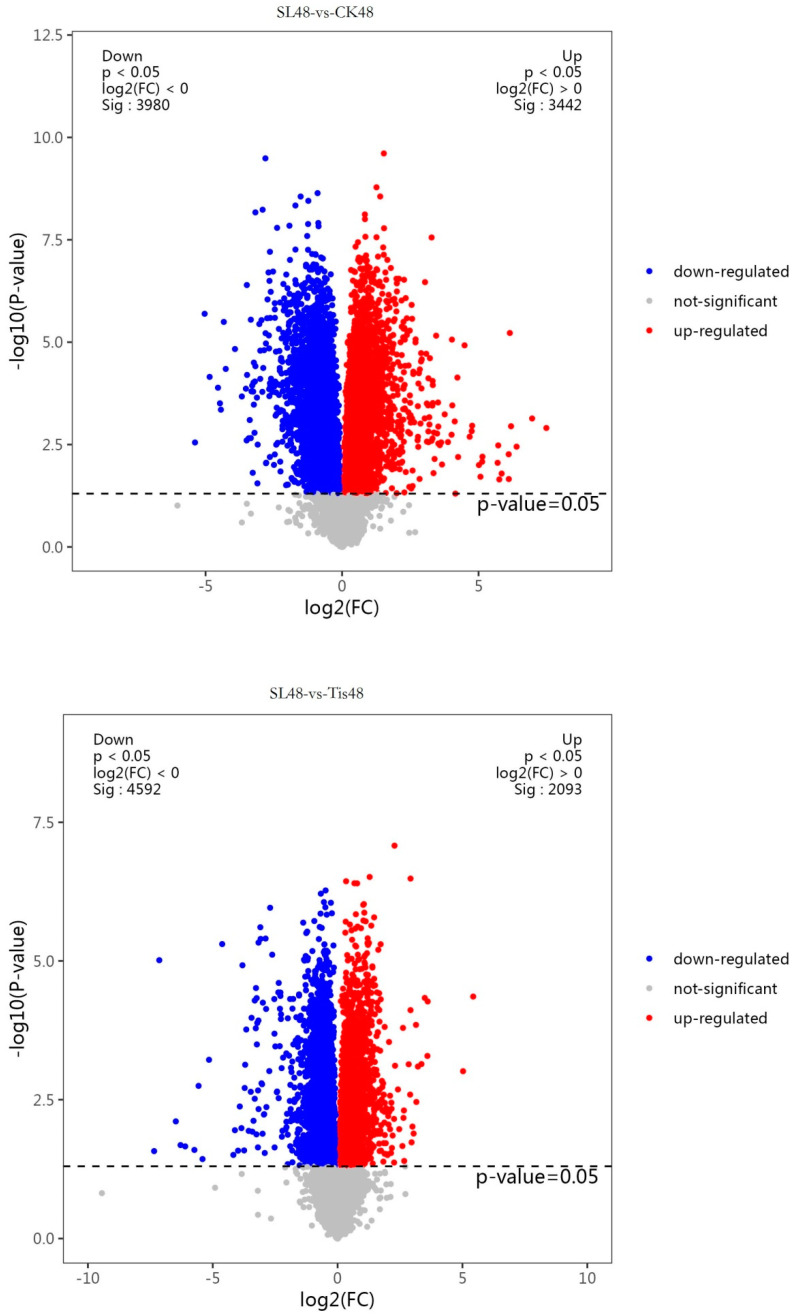

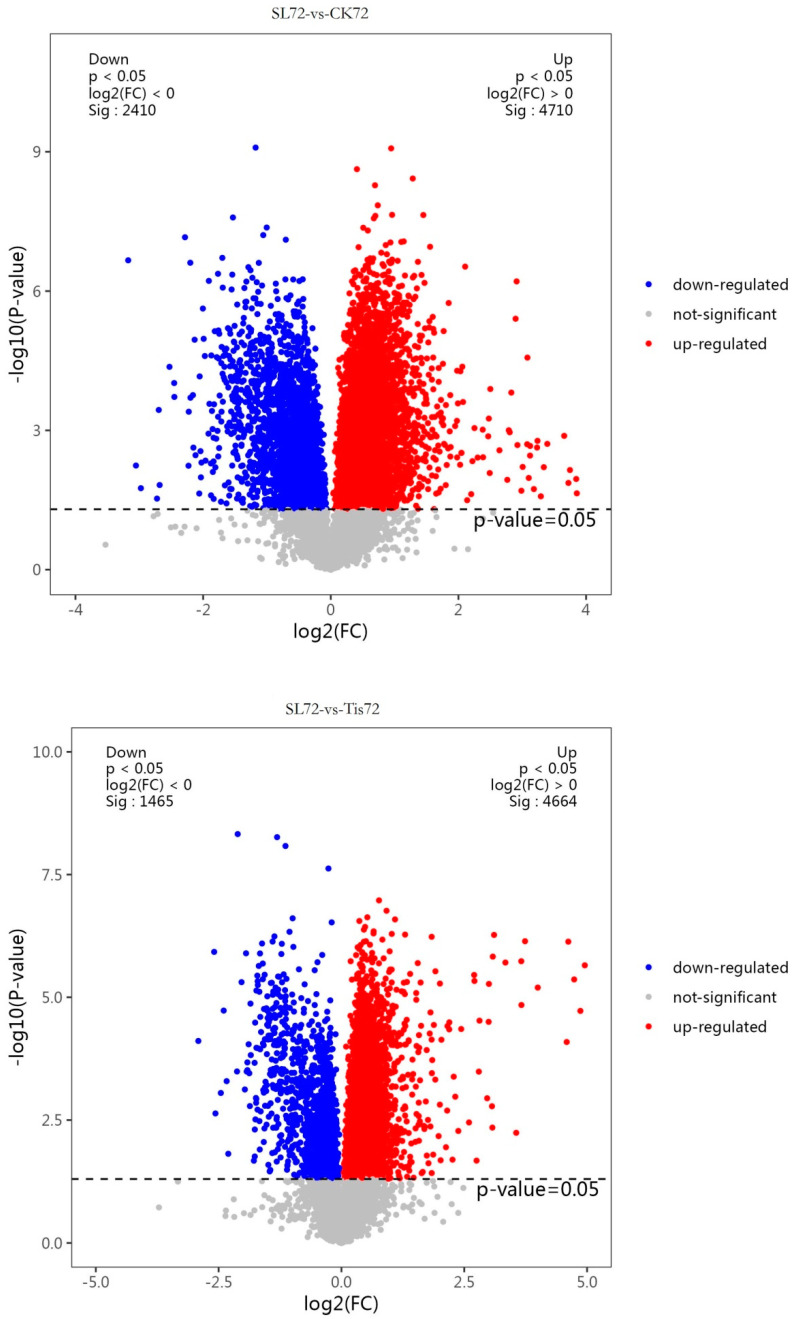

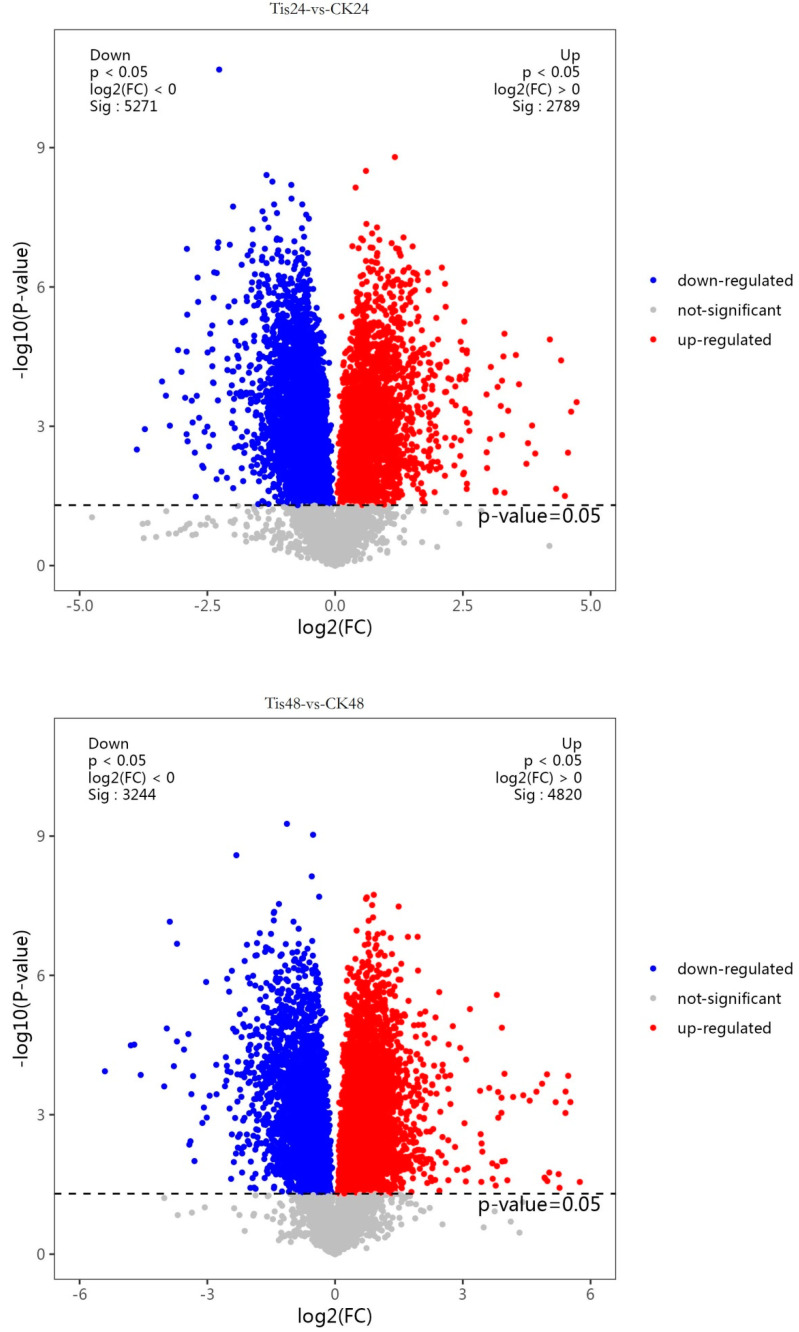

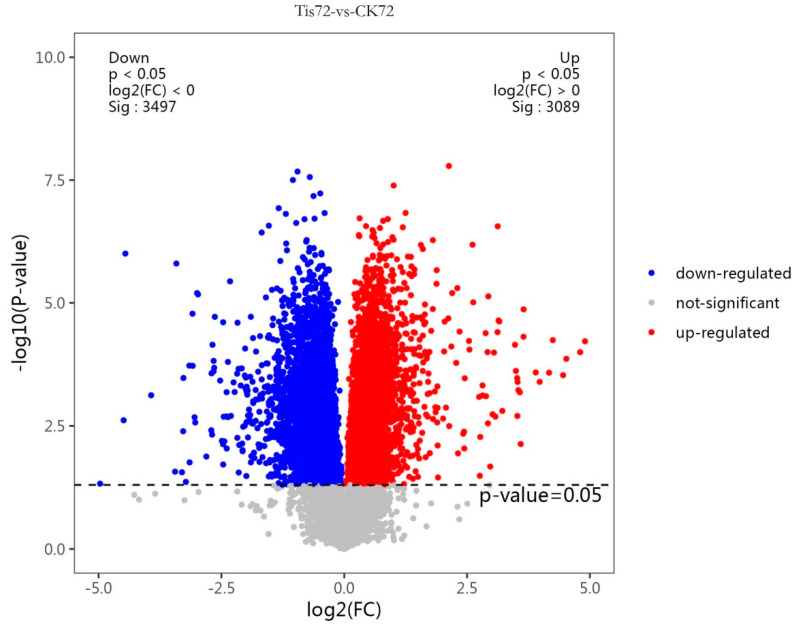
Metabolome volcano diagram. Remark: 24, 48, and 72 represent the sampling time after treatment with different drugs, SL represents treatment with *Strigolactone*, Tis represents treatment with *Strigolactone* inhibitor, and CK represents control treatment.

As shown in Figure 8, correlation analysis and least-square correlation analysis were carried out for different periods and different treatments, and the Q2Y value was 0.952, indicating that the adopted model was effective and reliable. Using *p* < 0.05 and log2 (FC) ≠ 0 as the standard to screen the differential metabolites, as shown in Figure 9, the differential metabolites in different periods and different treatments for 24 h (SL/ck increased by 4842 and decreased by 2763; Tis/CK increased by 5271 and decreased by 2789; SL/Tis increased by 2057 and decreased by 2685), 48 h (SL/CK increased by 3980 and decreased by 3442; Tis/CK increased by 3244 and decreased by 4820; SL/Tis increased by 4592 and decreased by 2093), and 72 h (SL/CK increased by 2410 and decreased by 4710; Tis/CK increased by 3497 and decreased by 3089; SL/Tis increased by 1465 and decreased by 4664) are shown.

**Figure 10:**
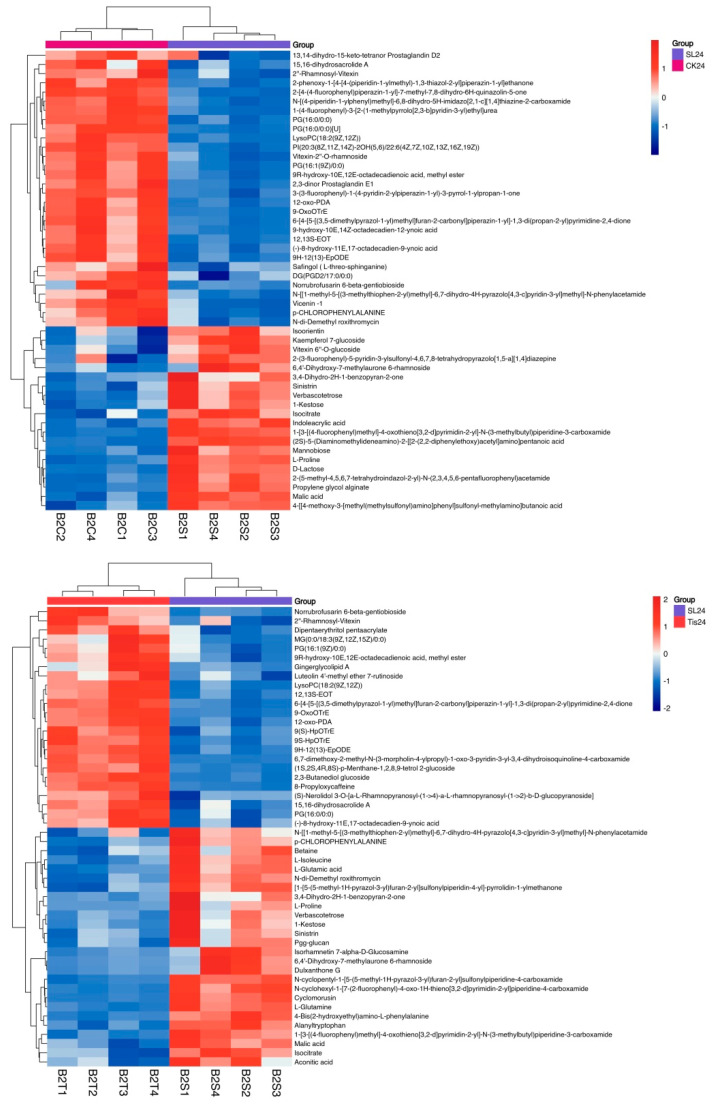

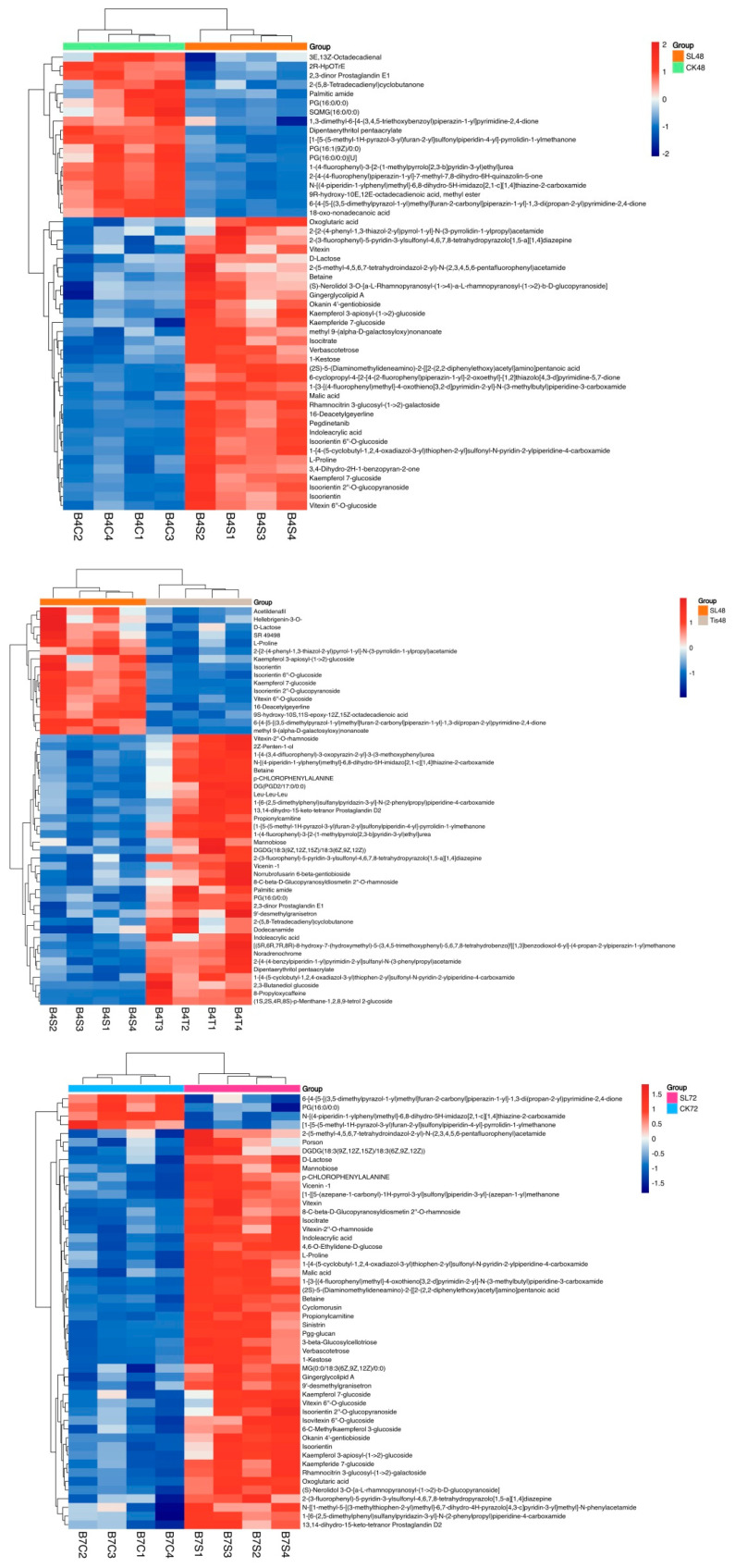

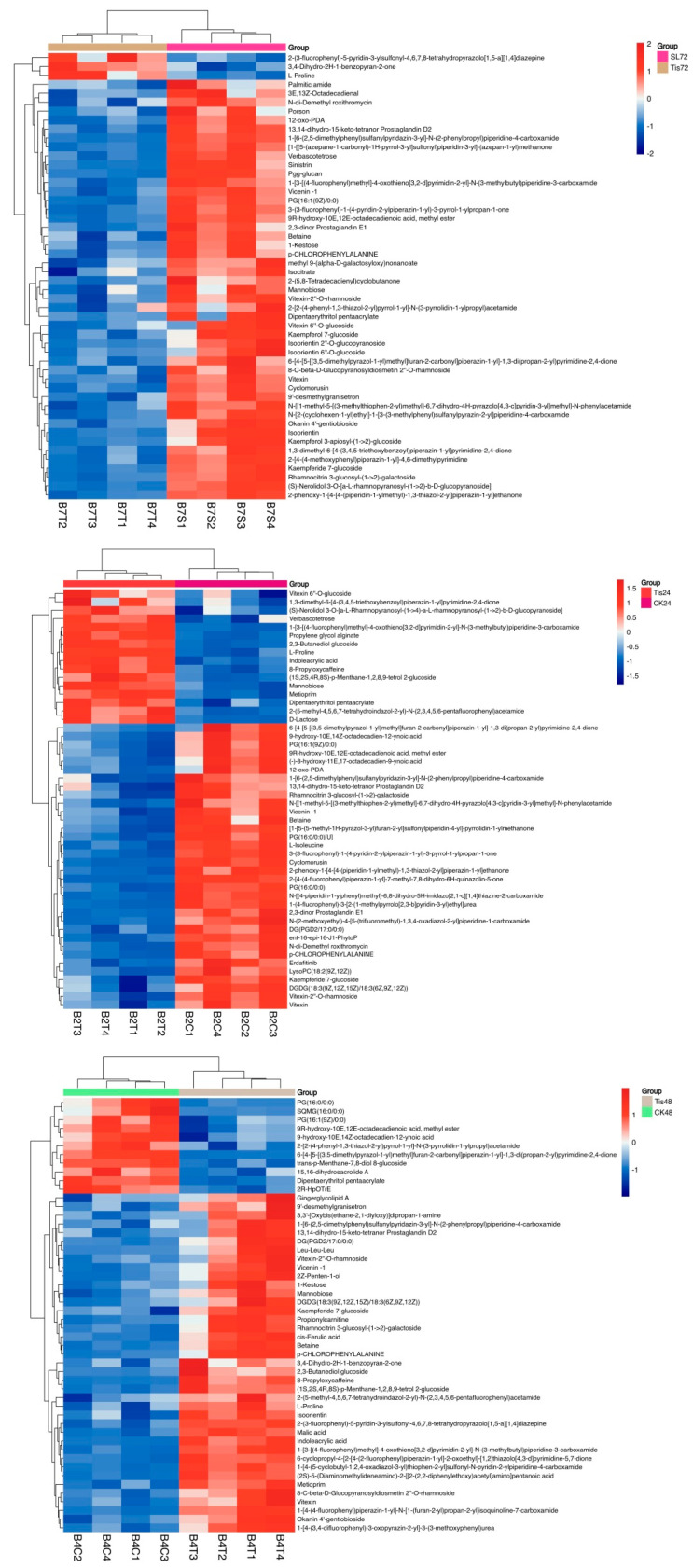

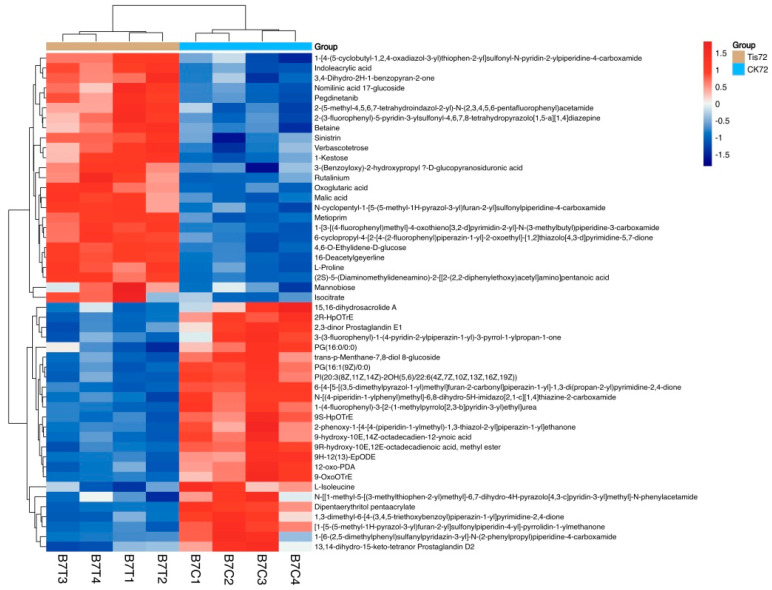
Metabolome clustering heatmap. Remarks: B2/B4/B7 represent sampling at 24 h, 48 h, and 72 h, respectively. C serves as the control (CK), representing water spraying. S represents treatment with one foot lactone (SL). T represents treatment with unicorn competing lactone inhibitors (Tis). Each treatment was repeated 4 times.

**Figure 11:**
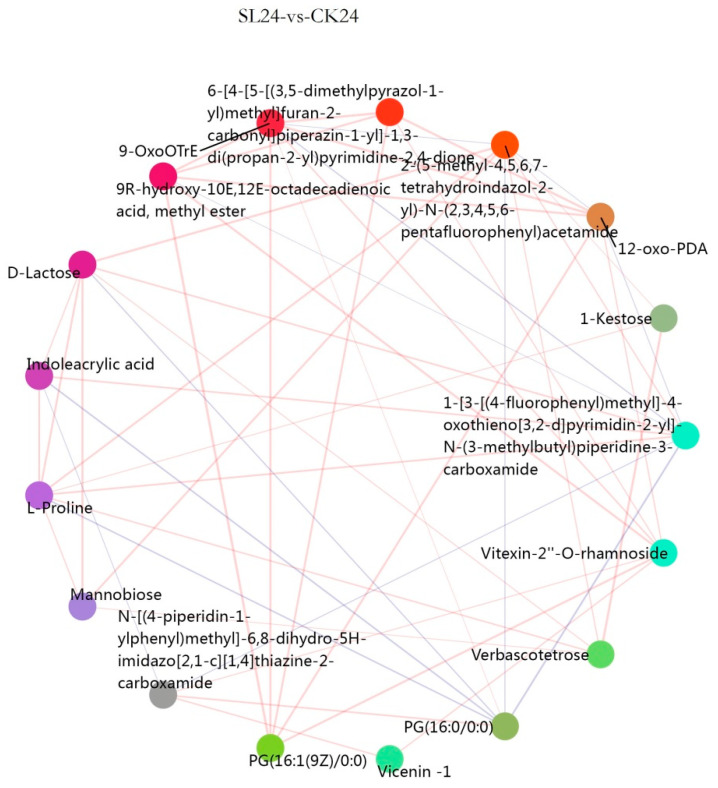

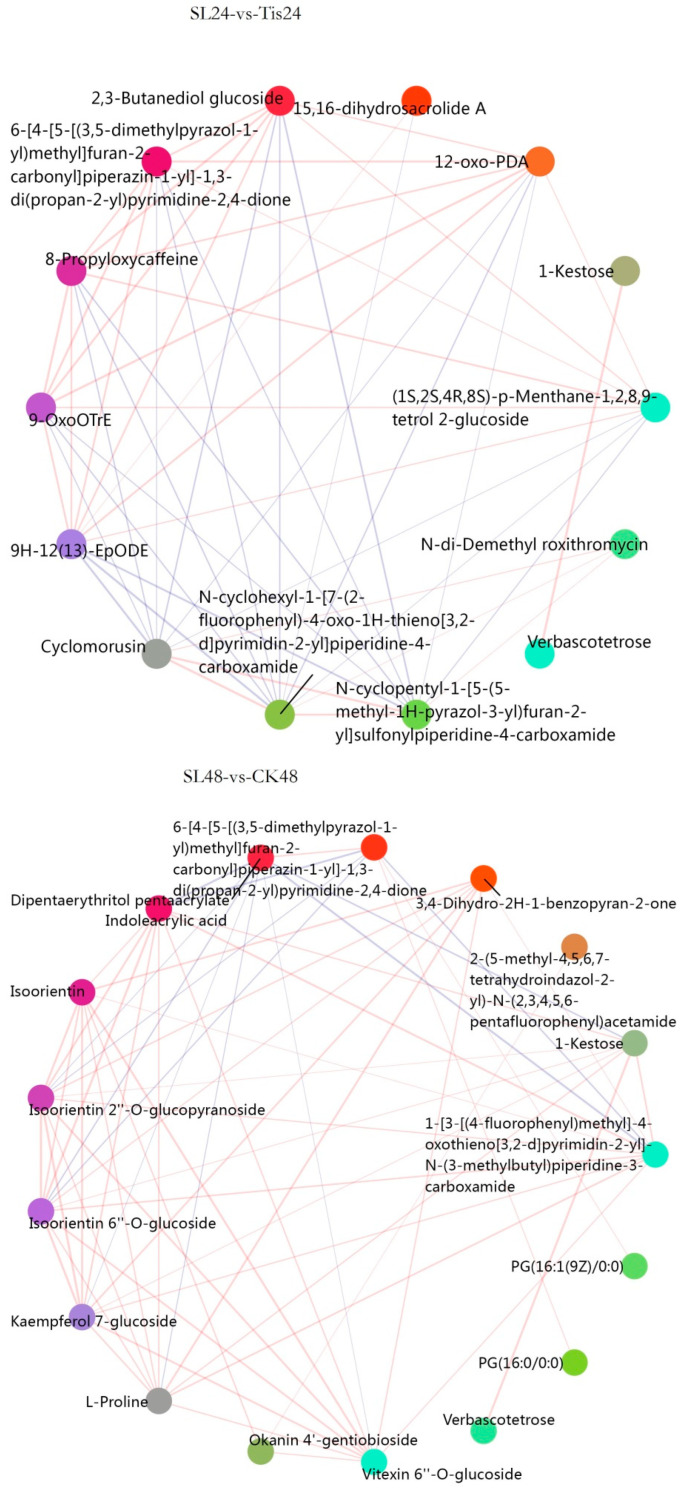

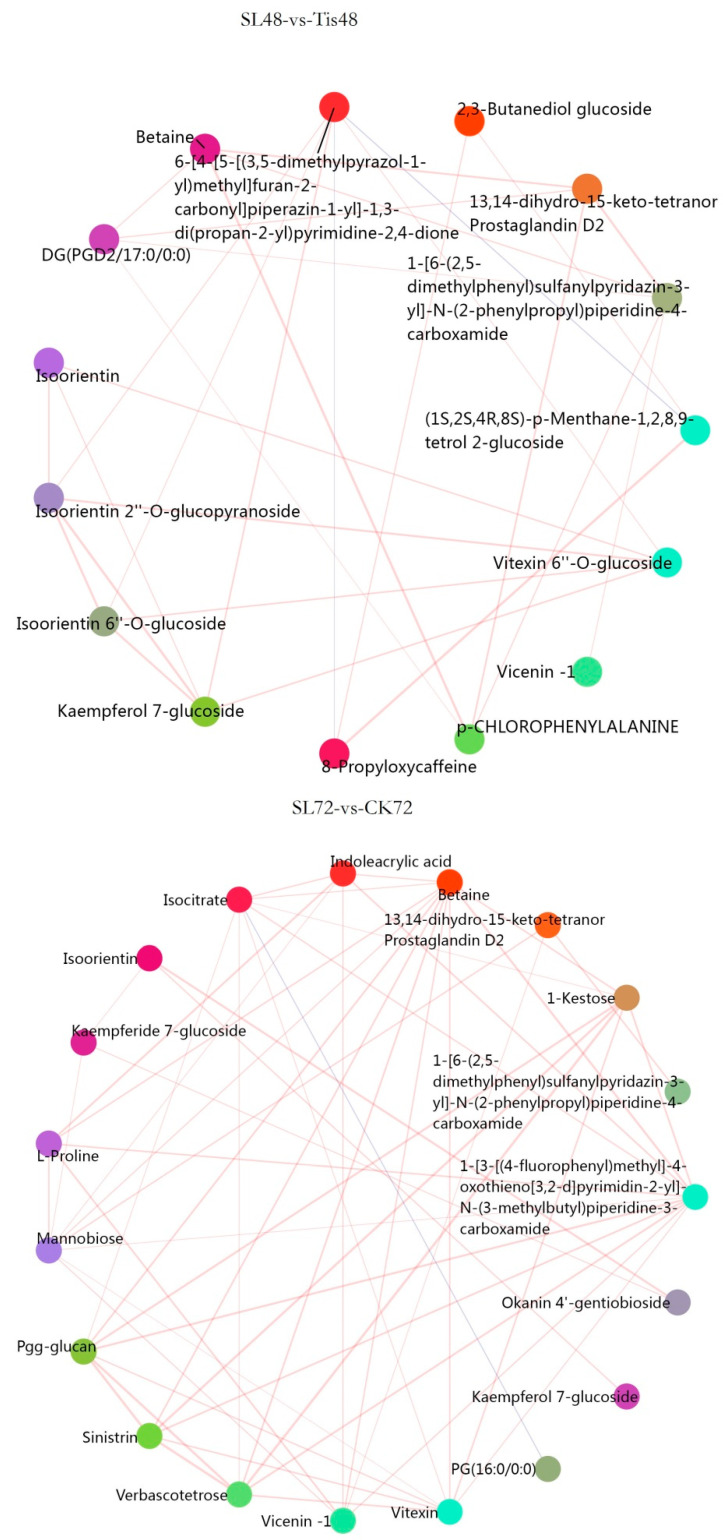

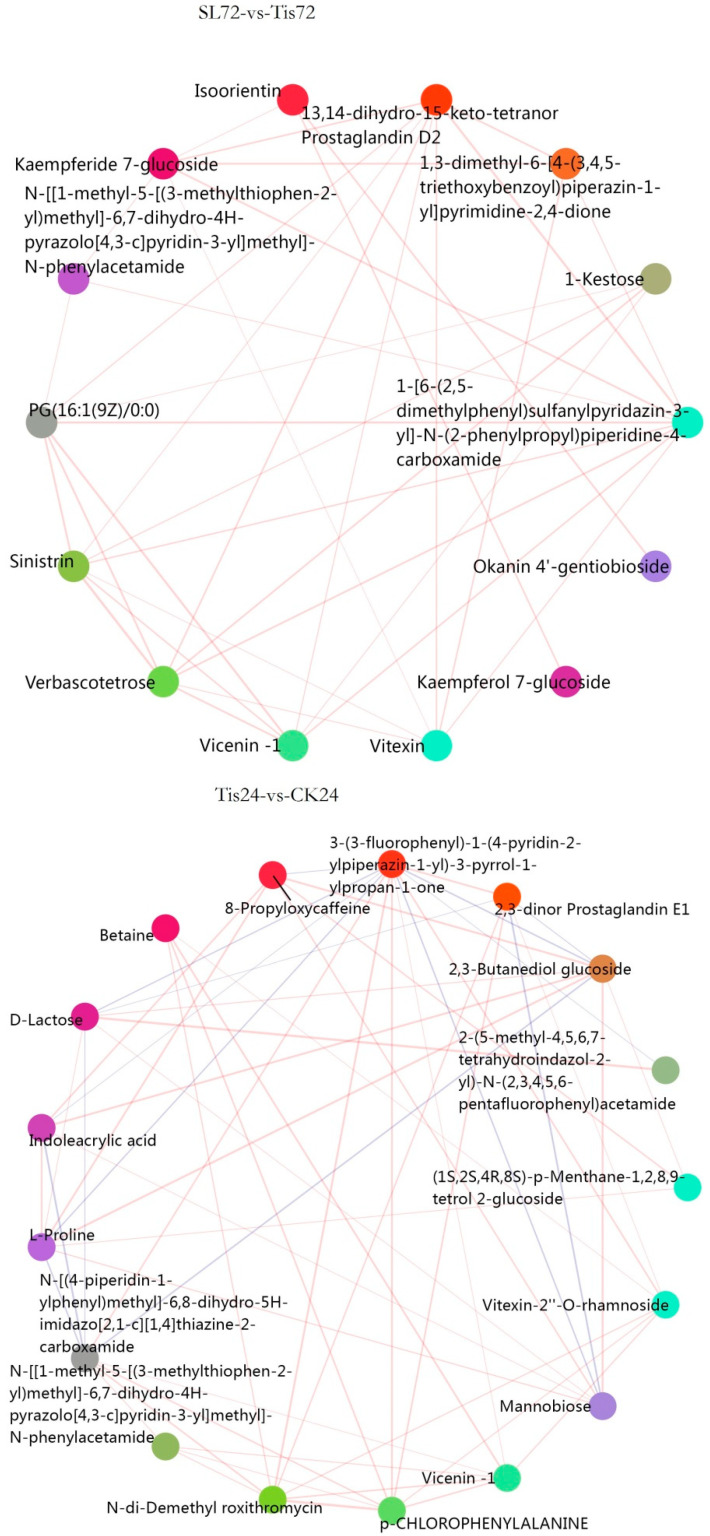

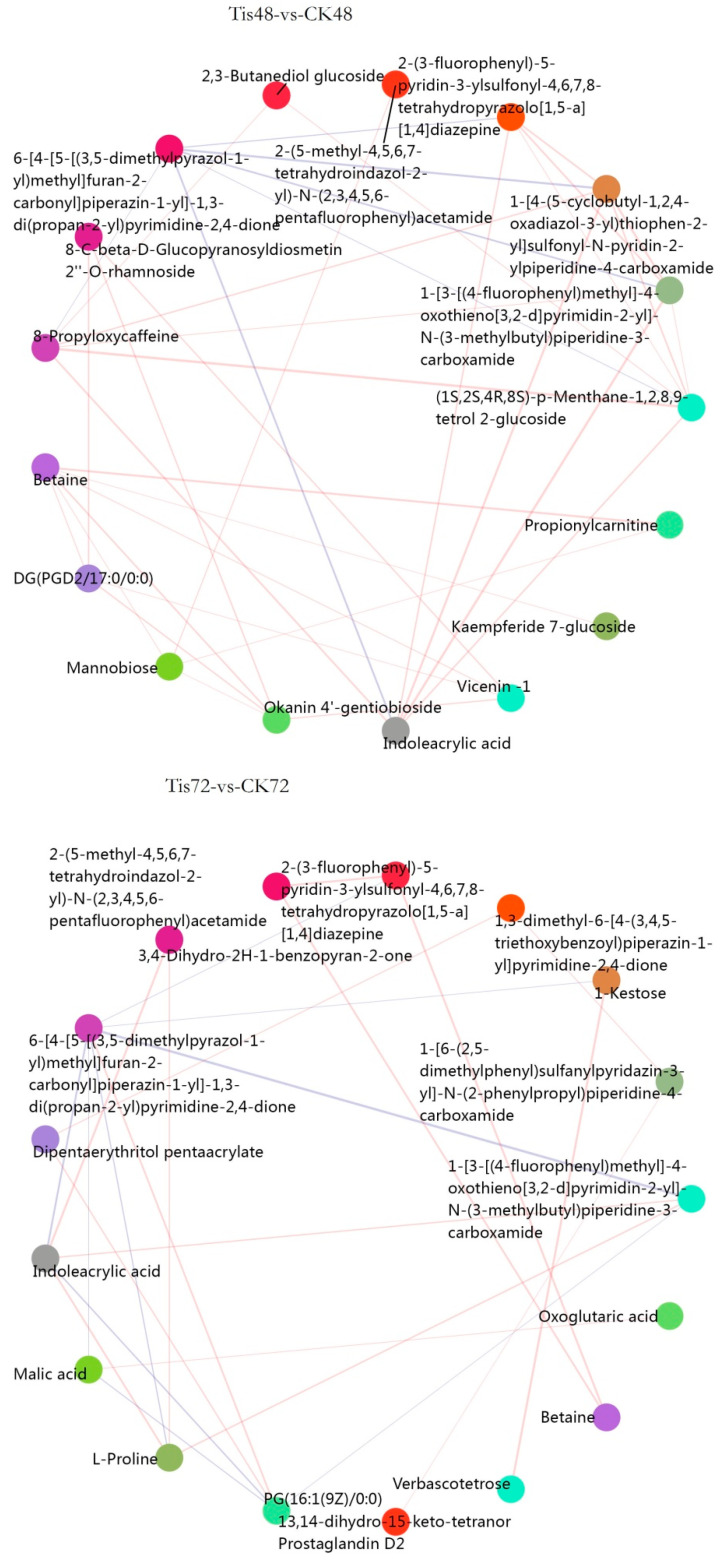
Metabolome correlation network diagram. Remark: 24, 48, and 72 represent the sampling time after treatment with different drugs, SL represents treatment with *Strigolactone*, Tis represents treatment with *Strigolactone* inhibitor, and CK represents control treatment.

The clustering effect of different treatments in different periods was clear, and the differences between groups were obvious (Figure 10), while the changes in differential metabolites were also obvious, including mannobiose, L-proline, D-lactose, malic acid, glucoside, isocitrate, indoleacetic acid, betaine, L-isoleucine, etc. According to the correlation analysis of metabolic pathways among different treatments (Figure 11), at 24 h (SL/CK proline, indoleacrylic acid, mannose, D-lactose, 1-ketose, etc.; Tis/CK betaine, mannobiose, proline and indoleacrylic acid, etc.; SL/Tis glucoside, 2,3-butanediol glucoside, 8-propoxycaffeine, etc.), 48 h (SL/CK proline, indoleacrylic acid, isoflavone glucoside, kaempferol glucoside, etc.; Tis/CK mannobiose, betaine, indoleacrylic acid, glucoside, etc.; SL/Tis glucoside, betaine, p-chlorophenylalanine, etc.), and 72 h (SL/CK indoleacrylic acid, isocitrate, mannobiose, glucose, etc.; Tis/CK pentaacrylic acid pentatriol, proline, betaine, indoleacrylic acid, etc.; SL/Tis glucoside, raffinose, etc.), they showed significant correlation changes in the differential metabolites.

## 3. Discussion

In this study, the research observed notable differences in the morphological development and physiological metabolism of wheat seedlings exposed to drought conditions, particularly when their leaves were sprayed with SL (salicylic acid) or Tis (an inhibitor of SL signaling). Compared to the control group (CK), wheat canopies treated with SL exhibited enhanced water retention capacity, reduced leaf bending, neater top leaves, and less canopy disorganization. Conversely, wheat treated with Tis displayed inhibited canopy growth and more dispersed parietal leaves. The analysis of physiological metabolism revealed that the SL-treated leaves showed heightened antioxidant enzyme activity and photosynthetic capacity, indicating that SL application can effectively bolster wheat seedlings’ drought tolerance. As an inhibitor of SL, Tis exerted a pronounced influence on wheat’s drought resistance.

Furthermore, utilizing second-generation sequencing technology, this study identified a multitude of genes expressed across various temporal stages and biological processes, with signal transduction genes primarily activated within 48 h. The transcription factor analysis pinpointed 20 factors, including single zinc finger binding proteins, that are abundantly enriched on chromosome 2. In conjunction with metabolome sequencing, the impacts of SL and Tis were primarily observed on glucosides, indoleacetic acid (IAA), betaine, mannose, and other compounds during the middle to late stages of wheat growth. These findings suggest that SL and Tis can influence wheat drought resistance, with particularly significant effects on IAA and brassinosteroid (BR) hormone metabolism, as well as osmoregulatory sugars. However, the mechanisms by which these transcription factors regulate wheat carbohydrate and hormone metabolism require further elucidation.

## 4. Materials and Methods

### 4.1. Study Settings

The test material was Zheng Mai 1860 which is a variety cultivated by the Wheat Research Institute of Henan Academy of Agricultural Sciences. Henan Academy of Agricultural Sciences is located in Zhengzhou, Henan Province, China. Henan Province is the most important agricultural province in northern China. Zheng Mai 1860 is a semi-winter variety with a relatively stable yield and good adaptability in most ecological regions in northern China. It is widely cultivated.

The seeds were sterilized and germinated to a plant height of about 10 cm, and uniform seedlings were selected for hydroponic culture. Hoagland nutrient solution was used for hydroponic culture, and it was changed every 2 days. Hydroponic seedlings were grown in an artificial climate box under environmental conditions (light/dark 12/12, brightness 20,000 lux, temperature 25/22 °C, humidity 50%). The seedlings were treated with drought stress when they grew to 2 leaves and 1 heart, that is, when the length of the top leaf was half of that of the middle leaf. An 18% PEG-6000 Hogland nutrient solution was used to simulate drought stress treatment (Hoagland nutrient solution was provided by coolebo company, Beijing, China).

After 48 h of stress treatment, the leaves were sprayed with Strigolactone (SLS; 3 μm/L), inhibitor (TIS; 10 μm/L), and acetone solution as the control (CK; 4.08 mm/L). The leaves were evenly covered with a layer of water film, and each treatment was repeated four times. The samples were taken at 24 h, 48 h and 72 h after spraying to determine the indicators. The experimental settings and codes are shown in Table 2.

### 4.2. Methods

Root and canopy material accumulation: at 72 h, two plants were randomly sampled from each pot, and the fresh weight of the root and canopy were weighed, respectively. After being killed at 105 °C for 30 min and dried at 85 °C to constant weight, they were weighed and the dry-to-fresh ratio was calculated.

Photosynthesis: SPAD and photosynthesis were measured at 24 h, 48 h, and 72 h.

Osmotic adjustment: the soluble sugar, protein, and free proline at 24 h, 48 h, and 72 h were determined.

Membrane lipid antioxidation: CAT, POD, SOD, and MDA at 24 h, 48 h, and 72 h were determined.

Joint analysis of transcriptome and metabolome: omics sequencing was completed by Shanghai OE Biotech Co., Ltd., China.

#### 4.2.1. Metabolome Screening and Analysis

First, 60 mg of the sample was weighed and put into a 1.5 mL centrifuge tube.

Two small steel balls and 600 μL of methanol water (*v*:*v* = 7:3, containing l-2-chlorophenylalanine, 4 μg/mL) were then added.

It was placed into a −40 °C refrigerator for 2 min of pre-cooling and then put into the grinder for grinding (60 Hz, 2 min).

Ultrasonic extraction was carried out in an ice water bath for 30 min, and it was left to stand at −40 °C overnight.

It was centrifuged at a low temperature for 10 min (12,000 rpm, 4 °C), 150 μL of supernatant was removed with a syringe, and then it was filtered with a 0.22 μm organic-phase pinhole filter, transferred to an LC injection vial, and stored at −80 °C until LC-MS analysis.

Quality control samples (QC) were prepared by mixing the extract of all samples in equal volume.

Remarks: All extraction reagents were pre-cooled at −20 °C before use.

A liquid chromatography–mass spectrometry system composed of a Dionex U3000 UHPLC ultra-performance liquid chromatography tandem QE plus high-resolution mass spectrometer was used (Thermo Fisher Scientific, Waltham, MA, USA).

Chromatographic column (Waters Corporation, Milford, MA, USA): ACQUITY UPLC HSS T3 (100 mm × 2.1 mm, 1.8 UM); column temperature: 45 °C; mobile phase: a-water (containing 0.1% formic acid), b-acetonitrile; flow rate: 0.35 mL/min; injection volume: 5 μL.

Ion source: ESI. The sample mass spectrum signal was collected in positive and negative ion scanning mode, respectively.

#### 4.2.2. Screening and Analysis of DOF Transcription Factors

RNA extraction and library construction: Trizol reagent was used to extract total RNA according to the instructions. RNA purity and quantification were identified using a nanodrop 2000 spectrophotometer (Thermo Scientific, USA), and RNA integrity was assessed using an Agilent 2100 Bioanalyzer (Agilent Technologies, Santa Clara, CA, USA). Transcriptome libraries were constructed using the VAHTS universal V6 RNA SEQ library prep kit according to the instructions. Transcriptome sequencing and analysis were performed by Shanghai Ouyi Biotechnology Co., Ltd., (Shanghai, China).

RNA sequencing and differential expression gene analysis: The library was sequenced using the Illumina novaseq 6000 sequencing platform (Illumina, Inc., San Diego, CA, USA), and 150 BP double-ended reads were generated. Approximately 69.54–86.24 raw reads were obtained for each sample. Fastp [48] software version 0.20.1 was used to process fastq format raw reads, and clean reads were obtained after removing low-quality reads for subsequent data analysis. Hisat2 [49] software version 2.2.1 was used for reference genome alignment, gene expression (fpkm) [50] was calculated, and the read counts of each gene were obtained by htseq count [51]. The PCA analysis and mapping of genes (counts) were performed using R (V 3.2.0) to evaluate sample biological replicates.

The differentially expressed genes were analyzed by deseq2 [52] software version 1.30.1, and the genes that met the threshold of Q value < 0.05 and FC > 2 or FC < 0.5 were defined as differentially expressed genes (DEGs). A hierarchical clustering analysis of DEGs was performed using R (V 3.2.0) to show the expression patterns of genes in different groups and samples. The R package ggradar was used to draw a radar map of the top 30 genes to show the expression changes of upregulated or downregulated genes.

Subsequently, GO pathway [53], KEGG pathway [54], reactome, and wikipathways enrichment analyses of differentially expressed genes based on a hypergeometric distribution algorithm were used to screen significant enrichment function items. R (V 3.2.0) was used to draw a column chart, chord chart, or enrichment analysis circle chart for significant enrichment function items.

## Figures and Tables

**Table 1:** DOF transcription factors.

Geneid	Description	Chromosome	Orientation
123064986	dof zinc finger protein DOF3.1-like	3B	minus
100415886	dof zinc finger protein DOF1.7	2A	minus
123127979	dof zinc finger protein 2-like	6A	minus
123190041	dof zinc finger protein 1-like	2A	plus
123137649	dof zinc finger protein 2-like	6B	plus
123046287	dof zinc finger protein 1-like	2B	plus
123104950	dof zinc finger protein DOF3.6-like	1B	minus
123187442	cyclic dof factor 1-like	2A	minus
123179992	dof zinc finger protein DOF3.6-like	1D	minus
123116001	dof zinc finger protein 3-like	5B	minus
123145076	dof zinc finger protein 2-like	6D	minus
123051520	cyclic dof factor 1-like	2D	minus
123186581	dof zinc finger protein DOF2.2-like	2A	minus
123188464	dof zinc finger protein DOF5.8-like	2A	minus
123044711	dof zinc finger protein DOF5.8-like	2B	minus
123052574	dof zinc finger protein DOF5.8-like	2D	minus
123093292	dof zinc finger protein DOF1.8-like	1B	plus
123040442	dof zinc finger protein DOF5.7-like	2B	plus
606390	dof zinc finger protein 4	6A	minus
100037553	dof zinc finger protein 4	6B	minus

**Table 2:** Experimental setup.

Time and Treatment	Transcriptome Number	Metabolome Number
24 h CK	R2C1	B2C1
24 h CK	R2C2	B2C2
24 h CK	R2C3	B2C3
24 h CK	R2C4	B2C4
24 h SL	R2S1	B2S1
24 h SL	R2S2	B2S2
24 h SL	R2S3	B2S3
24 h SL	R2S4	B2S4
24 h Tis	R2T1	B2T1
24 h Tis	R2T2	B2T2
24 h Tis	R2T3	B2T3
24 h Tis	R2T4	B2T4
48 h CK	R4C1	B4C1
48 h CK	R4C2	B4C2
48 h CK	R4C3	B4C3
48 h CK	R4C4	B4C4
48 h SL	R4S1	B4S1
48 h SL	R4S2	B4S2
48 h SL	R4S3	B4S3
48 h SL	R4S4	B4S4
48 h Tis	R4T1	B4T1
48 h Tis	R4T2	B4T2
48 h Tis	R4T3	B4T3
48 h Tis	R4T4	B4T4
72 h CK	R7C1	B7C1
72 h CK	R7C2	B7C2
72 h CK	R7C3	B7C3
72 h CK	R7C4	B7C4
72 h SL	R7S1	B7S1
72 h SL	R7S2	B7S2
72 h SL	R7S3	B2C1
72 h SL	R7S4	B2C2
72 h Tis	R7T1	B2C3
72 h Tis	R7T2	B2C4
72 h Tis	R7T3	B2S1
72 h Tis	R7T4	B2S2

## Data Availability

The data supporting the results of this study can be applied to the author and provided after being approved by the unit.
